# Artificial intelligence for women’s reproductive health: A scoping review of global diagnostic trends, methodological gaps, and a translational research agenda for low-resource settings

**DOI:** 10.1371/journal.pone.0358557

**Published:** 2026-09-25

**Authors:** Mohammad Mehedi Hasan Munna, Toriqul Islam, Omar Faruk, Reefka Fabliha Tulona, Afsin Sultana, Fairuj Saima, Acramul Haque Kabir, Tania Sultana, Raihan Ul Islam

**Affiliations:** 1 Department of Computer Science and Engineering, East West University, Dhaka, Bangladesh; 2 Institute of Science and Technology, National University Bangladesh, Dhaka, Bangladesh; 3 Department of Computer Science and Engineering, Independent University, Bangladesh (IUB), Dhaka, Bangladesh; 4 Department of Electrical and Computer Engineering, North South University, Dhaka, Bangladesh; Korea University - Seoul Campus: Korea University, KOREA, REPUBLIC OF

## Abstract

Women’s reproductive and endocrine disorders including Polycystic Ovary Syndrome (PCOS), endometriosis, thyroid disorders, infertility, and pregnancy-related complications remain a major global health burden. These conditions are especially difficult to manage in low- and middle-income countries (LMICs), where diagnostic facilities and specialist care are limited. We conducted a PRISMA-ScR–guided scoping review of artificial intelligence (AI) and machine learning (ML) applications in women’s reproductive and hormonal health. Our search covered five databases PubMed, Scopus, IEEE Xplore, Web of Science, and Google Scholar and included peer-reviewed studies published between 2021 and 2025. A total of 116 eligible studies were identified and classified into six categories: clinical prediction and risk modeling, medical imaging and AI, biomarker discovery and multi-omics, reproductive and endocrine health applications, clinical decision support systems, and AI/ML methodology development. Ensemble methods such as Random Forest and Gradient Boosting showed strong and consistent diagnostic performance. Convolutional neural networks performed well in single-centre settings but showed reduced performance upon external validation, consistent with optimism bias. Overall, 83.6% of studies were classified as high risk of bias and 75.5% lacked external validation. After adjusting for population size, high-income countries produced 23 times more studies per million women of reproductive age than LMICs. Sub-Saharan Africa contributed fewer than 0.1 studies per million women, and no studies were identified from Nepal or Sri Lanka. Current evidence, which is heavily concentrated in high-income and East Asian settings, is insufficient to support population-level deployment. We introduce a four-tier LMIC feasibility classification system that links data modality, infrastructure requirements, and personnel needs. We also propose a phased research agenda that separates near-term priorities (0–2 years) from medium-term goals that depend on infrastructure investment.

## 1. Introduction

Women’s reproductive and hormonal disorders constitute a significant yet persistently underaddressed global health challenge, with profound implications for fertility, metabolic function, mental health, and quality of life [1–3]. Reproductive and hormonal disorders are complex conditions resulting from a mix of genetic, environmental and lifestyle factors [1,4,5]. Although these are very common, they are highly under-diagnosed and under-treated, resulting in considerable health burden, sub-fertility, disability-adjusted life years (DALYs), and negative health outcomes in later life, such as cardiovascular and metabolic diseases and psychological disturbances [2,5]. PCOS affects 5–18% of women of reproductive age, and is closely associated with a number of other important comorbidities, such as insulin resistance, obesity, dyslipidaemia and depression [1,4,6]. Endometriosis affects 5–10% of women, with diagnostic delays often exceeding 8–12 years [7,8]. Women’s health is a systemic domain where women’s symptoms are often normalised or dismissed [7–9], and hence inadequate care is a common issue affecting these disorders.

A huge burden of reproductive and hormonal disorders also exists in low- and middle-income countries (LMICs) in South Asia, Sub-Saharan Africa, and South East Asia, where there is a coexistence of socio-economic burden, absence of specialist healthcare services, limited diagnostic capacity, and societal stigma [10]. In Bangladesh, the Maternal Mortality Ratio (MMR) has been reported to decrease from 323 deaths/100,000 live births in 2001–116 deaths/100,000 live births in 2017, but non-acute reproductive and hormonal disorders are not optimally managed. The prevalence of PCOS in Bangladesh has been estimated to be 7.8–20% as per the Rotterdam criteria, and diagnostic delays of over two years have been reported in rural Bangladesh [10,11]. Rapid expansion in mobile phone ownership and internet connectivity across LMICs provides a viable foundation for AI-enabled health solutions, yet pilot studies including work on early gestational diabetes prediction using wearable devices and explainable AI in South Africa highlight the gap between technological readiness and clinical integration [10,11].

**Fig 1:**
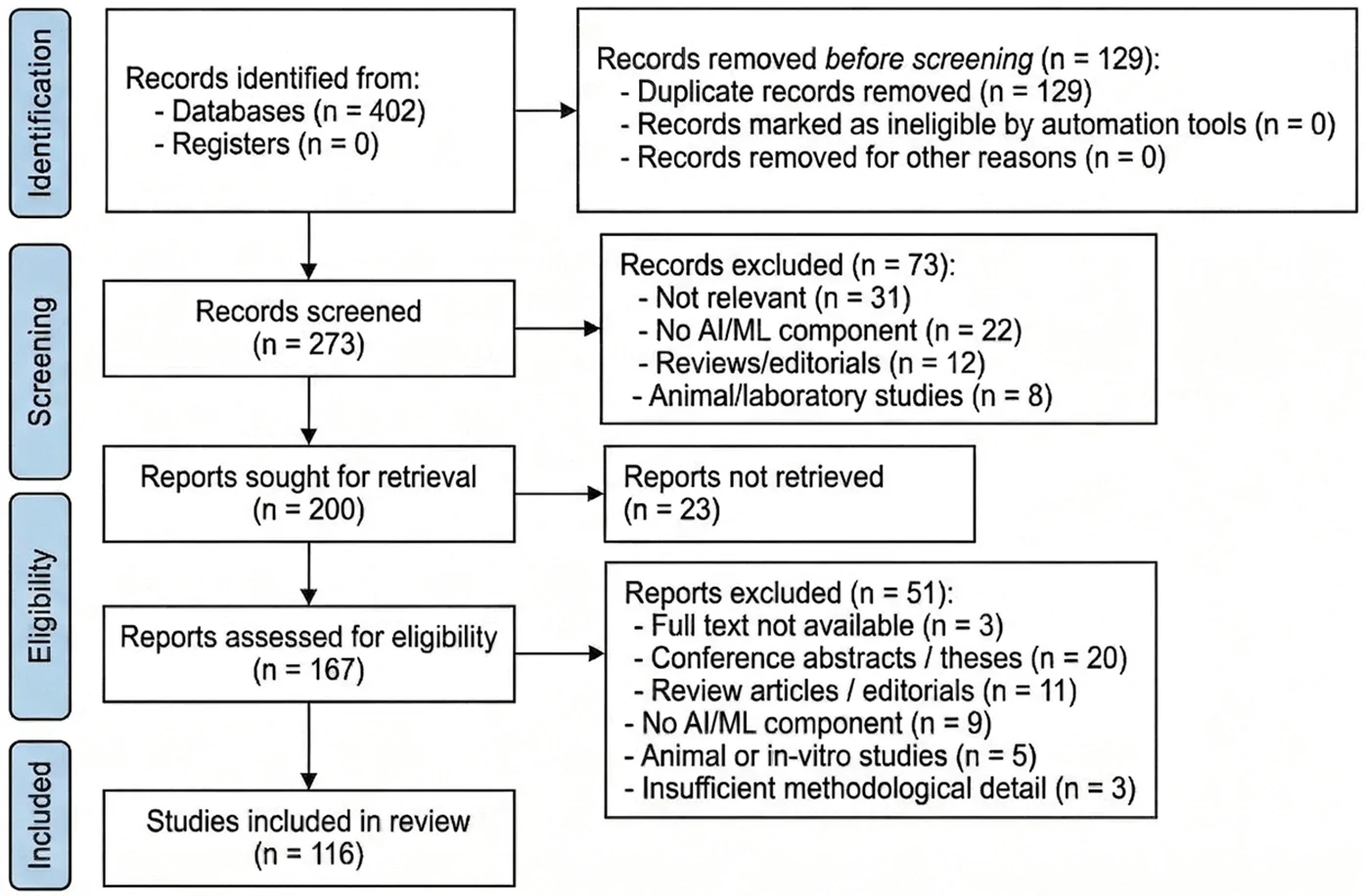
PRISMA flow diagram. Illustrating the study identification, screening, eligibility assessment, and inclusion process.

Artificial intelligence (AI) and machine learning (ML) have shown strong potential in healthcare. They are particularly useful for pattern recognition, risk prediction, and clinical decision support. These methods have been applied to many types of data, including clinical records, hormonal profiles, medical imaging, and lifestyle indicators. Studies have reported high diagnostic performance for conditions such as PCOS, endometriosis, and gestational diabetes [12–16]. However, most existing models have important limitations. They were developed in high-income countries using small or single-centre datasets [12,14]. Many lack external validation or interpretability assessment. This raises serious concerns about whether these models can work reliably in low- and middle-income country (LMIC) settings [10]. No existing review has systematically examined methodological trends and translational readiness from an LMIC perspective. Consequently, the findings of this review reflect trends predominantly from East Asian and high-income settings and should not be interpreted as globally generalizable trends. Translational recommendations for LMICs remain theoretical until validated in local, resource-constrained populations. Some recent reviews have addressed related topics. Mengistu et al. [15] conducted a broad scoping review of AI in sexual and reproductive health. Ghaderzadeh et al. [12] systematically reviewed AI applications specifically in PCOS. However, neither review examined methodological rigor, resource-stratified deployment feasibility, and geographic equity together. These three factors are essential for assessing translational readiness in LMIC settings. The present review addresses this gap through three key contributions. First, it proposes a six-category methodological taxonomy covering all major reproductive and endocrine conditions. Second, it introduces a four-tier LMIC feasibility classification system that links data modality, infrastructure, and personnel requirements. Third, it presents a population-adjusted geographic equity analysis. This analysis reveals significant research voids in Nepal, Sri Lanka, and most of Sub-Saharan Africa [10,11]. This paper presents a PRISMA-ScR–guided scoping review of AI and ML applications in women’s reproductive and hormonal health (Fig 1). It covers studies published between 2021 and 2025. The review focuses explicitly on translational applicability in low-resource settings. Its goal is to support the development of robust, equitable, and context-aware AI solutions for LMICs [10,12].

## 2. Materials and methods

This scoping review followed the PRISMA-ScR guidelines (Preferred Reporting Items for Systematic Reviews and Meta-Analyses Extension for Scoping Reviews) [17]. PRISMA-ScR includes 20 essential items and 2 optional items. The completed checklist is provided as S1 File. These guidelines were chosen to make the review transparent, reproducible, and complete. This was especially important because the review aimed to explore and map existing literature. A predefined protocol guided the entire review process. It covered all major stages: literature search, screening, eligibility assessment, data extraction, study classification, and synthesis. Since this is a scoping review rather than an intervention-based systematic review, formal registration in PROSPERO was not required. However, the protocol was developed before data extraction began and was deposited in the Mendeley Data repository [18]. The repository includes screening metadata for all 273 identified records. It contains the reasons for exclusion at both the first and second screening stages. It also includes final inclusion decisions for the 116 eligible studies and their taxonomy classifications. This was done in accordance with the PRISMA 2020 data sharing guidelines.

### 2.1 Research questions

The research questions were formulated using the *PICO* (Population, Intervention, Comparison, Outcome) framework [19]. The population comprised women affected by reproductive and hormonal disorders; the intervention included AI and ML–based diagnostic or predictive approaches; comparisons involved conventional or non–AI-based strategies where applicable; and outcomes focused on diagnostic accuracy, risk prediction, clinical decision support, and translational feasibility in low-resource healthcare settings. The review was guided by the following research questions:

- **Q1:** What are the clinical applications of AI and ML in women’s reproductive and hormonal health?
- **Q2:** Which AI/ML tasks and models are most frequently used across different reproductive and endocrine disorders?
- **Q3:** What data modalities are employed to develop AI-based diagnostic and predictive systems?
- **Q4:** How are existing studies distributed across disease-specific and methodological taxonomies, and what translational gaps exist for low-resource healthcare settings?

### 2.2 Search strategy

A comprehensive literature search was conducted across five major academic databases: *PubMed, Scopus, IEEE Xplore, Web of Science, and Google Scholar*. All searches were performed between *December 2025 and February 2026*, and results were date-stamped to ensure reproducibility. The search strategy was designed to identify peer-reviewed studies applying artificial intelligence, machine learning, or deep learning techniques to women’s reproductive and hormonal health. Search terms were organized into two conceptual domains and combined using Boolean operators (AND/OR). The complete database-specific search queries are summarized in Table 1. The 2021 start date was selected because it marks the inflection point where transformer-based architectures and the post-COVID acceleration of digital health research substantially reshaped the AI-in-healthcare literature, rendering pre-2021 methodological surveys insufficient for characterizing the current state of the field. Earlier work is well covered by previous reviews [12,15,16]. To ensure full reproducibility, we explicitly restricted the search to English-language publications. Grey literature, including preprints, unpublished theses, and conference abstracts without full peer-reviewed papers, were explicitly excluded to ensure the inclusion of only methodologically complete, validated studies. The complete, database-specific search strings, including all Boolean operators and field tags, are provided in Table 1.

**Table 1:** Database-specific search parameters.

Database	AI/ML Terms	Health Domain Terms	Field Tags	Date Range
PubMed	“artificial intelligence”, “machine learning”, “deep learning”	“women’s health”, “PCOS”, “endometriosis”, “thyroid”, “reproductive disorder”, “hormonal disorder”, “menstrual disorder”	[TIAB]	2021–2025
Scopus	Same as above	Same as above	TITLE-ABS-KEY	2021–2025
Web of Science	Same as above	Same as above	TS=	2021–2025
IEEE Xplore	Same as above	Same as above	All Metadata	2021–2025
Google Scholar	Same as above	“women’s reproductive health”, “PCOS”, “endometriosis”, “infertility”, “menstrual disorder”, “thyroid”	Full-text	2021–2025

All term groups combined using Boolean OR within each concept block and AND between blocks. Searches restricted to English-language peer-reviewed articles and conference proceedings. Google Scholar: first 200 results screened per query. All searches were conducted between December 2025 and February 2026.

### 2.3 Eligibility criteria

Studies were considered eligible if they satisfied all of the following criteria:

- Original peer-reviewed research articles;
- Application of AI, ML, or deep learning techniques;
- Focus on women’s reproductive or hormonal disorders;
- Use of human clinical, imaging, biochemical, lifestyle, or multimodal data;
- Publication in English between January 1, 2021 and December 31, 2025.

Studies were excluded if they met any of the following conditions: review articles, meta-analyses, editorials, commentaries, or book chapters; conference abstracts without full papers; animal or laboratory-based studies; studies unrelated to reproductive or endocrine health; absence of an AI/ML methodological component; or inaccessible full text or insufficient methodological detail.

### 2.4 Study selection process

All retrieved records were exported into Microsoft Excel, and duplicate entries were removed using automated deduplication followed by manual verification. Study selection was conducted using a predefined two-stage screening process managed through Rayyan AI [20,21] screening software. In the first stage, titles and abstracts were independently screened by two reviewers (MMHM and TI) to assess relevance to the research questions. Studies deemed irrelevant by both reviewers were excluded, while discrepancies were resolved through discussion with a third reviewer (TS). In the second stage, full-text articles of potentially eligible studies were independently assessed against the predefined eligibility criteria by two reviewers (AFS and FS). Reasons for exclusion at this stage were documented using a standardized classification system. Initial database searches identified 402 records. After removing 129 duplicates, 273 records underwent title/abstract screening. Of these, 167 proceeded to full-text assessment after excluding 73 records at title screening and 23 at abstract screening. Full-text review of 167 articles resulted in a further 51 exclusions, yielding 116 studies that satisfied all inclusion criteria for final synthesis (Fig 1). Of the 116 final studies, 94 (81.0%) were retrieved from PubMed, Scopus, IEEE Xplore, or Web of Science, and 22 (19.0%) were unique to Google Scholar. The Google Scholar 200-result limit therefore affected approximately one-fifth of the final corpus, and sensitivity to this cap was assessed by cross-checking citation overlap across databases.

### 2.5 Data extraction and charting

Data extraction was performed using a standardized Excel-based data-charting form, which was iteratively refined during a pilot phase involving 10 randomly selected studies. For each included study, data were extracted across six specific domains:

1. **Bibliographic information** (database source, title, authors, publication year, journal/conference);
2. **Study characteristics** (research objective, target disease/condition, study design, sample size, geographic region, funding source);
3. **Data modalities** (clinical records, hormonal/biochemical markers, medical imaging, lifestyle/demographic data, multimodal integration);
4. **AI/ML components** (tasks, algorithms employed, software/implementation details);
5. **Performance metrics** (accuracy, sensitivity, specificity, AUC, F1-score, validation methods); and
6. **Translational indicators** (data accessibility, computational requirements, cost considerations, ethical considerations, deployment feasibility in LMICs).

A complete data extraction dictionary detailing the operational definitions for all extracted variables is provided in the supplementary repository.

To ensure high fidelity and minimize extraction errors, the data underwent a rigorous, multi-tier independent verification process. Initial data extraction was conducted by one reviewer (TI). In the second stage, the extracted data were independently verified by three reviewers (OF, AFS, and FS). Subsequently, a third verification was conducted by MMHM to ensure comprehensive accuracy across the entire dataset.

A 20% random sample of the fully verified data was cross-verified, with inter-rater agreement calculated using Cohen’s κ (κ=0.92). Any remaining discrepancies at any stage of the verification process were resolved through discussion and consensus with the senior authors (RUI and TS).

### 2.6 Study classification and taxonomy

The classification taxonomy was developed through an iterative three-step process: (1) initial framework based on prior AI-in-healthcare reviews [12,15], (2) pilot testing and refinement using 20 randomly selected studies, and (3) finalization through team consensus meetings involving all authors. Each study was assigned to a single primary taxonomy category based on its principal research objective, data modalities, and AI/ML tasks. In cases where studies addressed multiple domains, classification was based on the primary contribution of the work. The final taxonomy comprised six categories: (1) Clinical Prediction and Risk Modeling; (2) Medical Imaging and Artificial Intelligence; (3) Biomarker Discovery and Multi-Omics Analysis; (4) Reproductive and Endocrine Health Applications; (5) Clinical Decision Support and Digital Health Tools; and (6) AI/ML Methodology and Algorithmic Development. To resolve boundary cases between Category 2 and Category 4, the following decision rule was applied prospectively: Category 2 was assigned when the primary methodological contribution was an imaging architecture or segmentation method; Category 4 was assigned when the primary contribution was clinical integration of AI with reproductive health data, regardless of whether imaging was involved. Studies combining ultrasound features with clinical parameters without proposing a novel imaging methodology were classified as Category 4.

### 2.7 Data synthesis and reporting

To ensure transparency in all descriptive summaries, the total number of included studies (N = 116) serves as the primary denominator for all reported counts, proportions, and percentages throughout the results, unless a specific subgroup denominator (e.g., studies specific to a single disease domain or algorithm) is explicitly stated in the corresponding table or text.

Given the heterogeneity in datasets, disease domains, and evaluation metrics across included studies, quantitative meta-analysis was not performed. Three structural violations precluded valid pooling: first, substantial clinical heterogeneity across disease domains (PCOS, endometriosis, thyroid, GDM) means that a pooled AUC estimate would constitute an ecological fallacy; second, 31 of 116 studies report multiple algorithms on identical datasets, violating the independence assumption required for meta-analytic pooling; third, overlapping training cohorts from shared public repositories (Kaggle PCOS, UCI thyroid) further compound statistical dependence. Formal heterogeneity testing (I²) was therefore not performed, as the independence violations render such statistics uninterpretable. This decision is consistent with established scoping review methodology, which prioritises breadth of evidence mapping over quantitative synthesis. All performance summaries are presented as descriptive ranges to characterise the reviewed literature, in alignment with scoping review methodology. Although descriptive AUC ranges are reported to characterise central tendency within algorithm categories, several structural threats to validity must be acknowledged: (1) ecological fallacy, as study-level AUC estimates aggregate outcomes across different diseases, populations, and imaging modalities; (2) statistical dependence, as 31 of 116 studies report multiple algorithms on identical datasets, violating independence assumptions; and (3) overlapping training cohorts from shared public repositories (e.g., UCI thyroid, Kaggle PCOS datasets). Given these violations, no inferential statistical testing (p-values, t-tests, or significance claims) is reported, and all quantitative summaries in Tables 4 and 5 are presented as descriptive approximations; no causal or superiority claim should be inferred.

Extracted data were summarised descriptively and thematically in relation to the four research questions: (1) clinical applications by disease domain, (2) AI/ML task and model distribution, (3) data modality trends, and (4) taxonomy-based patterns with translational implications. Descriptive AUC ranges were compiled from author-reported metrics for each algorithm category.

### 2.8 Risk-of-bias and quality assessment

The included corpus spans four methodologically distinct study types — diagnostic imaging, clinical prediction modeling, biomarker discovery, and algorithm development — each governed by a different validated risk-of-bias instrument (QUADAS-AI for imaging, PROBAST-AI for prediction, and TRIPOD-AI reporting standards for algorithm development). Item-by-item application of any single instrument across this heterogeneous corpus would have produced systematic misclassification, because items relevant to one study type are inapplicable to another. To preserve cross-corpus comparability while retaining domain-specific validity, we adopted a harmonized five-domain framework synthesizing the shared core items of PROBAST-AI, QUADAS-AI, and TRIPOD-AI. Each study was evaluated across five domains: (1) participant selection and data representativeness; (2) predictor measurement; (3) outcome assessment and ground truth reliability; (4) model development and validation strategy; and (5) reporting transparency. Studies were categorized as having *low*, *moderate*, or *high* risk of bias based on cumulative assessment across domains. The complete scoring rubric, including explicit criteria for low- and high-risk classification in each domain, is provided in S1 Table. Studies meeting high-risk criteria in 1–2 domains were classified as moderate risk; studies meeting high-risk criteria in 3 or more domains were classified as high risk. The number of studies at each risk level per domain is reported in Table 10.

### 2.9 LMIC applicability assessment

Each included study was evaluated for deployment feasibility in LMIC settings using a four-tier qualitative classification scheme, defined prospectively based on infrastructure requirements, personnel needs, cost per test, and appropriate care level. The four categories are: (1) High Feasibility — requires only basic clinical variables, no specialist personnel, cost < $10/test, deployable in primary care; (2) Moderate Feasibility — requires routine laboratory tests or basic ultrasound, nurse- or midwife-level personnel, cost $10–25/test, deployable in secondary care; (3) Low Feasibility — requires specialist imaging or advanced laboratory panels, specialist personnel, cost $25–50/test, requires district hospital; and (4) Very Low Feasibility — requires MRI or advanced imaging or multi-omics, highly specialized personnel, cost > $50/test, requires tertiary centre only. The full categorical criteria are provided in S2 Table. This qualitative scheme was adopted in preference to a continuous numeric score to avoid conveying unwarranted precision and to ensure reproducibility of classification across reviewers.

### 2.10 Limitations of the methodology

- **Language restriction:** Inclusion of only English-language publications may exclude relevant studies from non-English speaking LMICs.
- **Database coverage:** While five major databases were searched, some relevant studies in regional or specialized databases may have been missed.
- **Grey literature exclusion:** Preprints, theses, and conference proceedings without full papers were excluded, potentially missing recent innovations.
- **Rapid field evolution:** The AI/ML field evolves quickly, and some advances from late 2025 may not be fully captured.
- **Scoping nature:** As a scoping review, detailed quality assessment of individual studies was not the primary focus, though general methodological trends were assessed.

### 2.11 Sensitivity analysis for overlapping public datasets

Several included studies draw on identical publicly available repositories, including the Kaggle PCOS dataset and the UCI thyroid dataset. To assess sensitivity of descriptive AUC ranges to dataset duplication, a collapsed dataset-level analysis was performed. Studies sharing a common training source were treated as a single observation for descriptive pooling purposes. After collapsing, effective independent dataset counts were:

- **PCOS (Kaggle-derived):** 20 studies collapsed to 11 independent datasets;
- **Thyroid (UCI-derived):** 24 studies collapsed to 14 independent datasets.

Collapsed descriptive AUC ranges shifted by less than 0.02 units across all algorithm families, suggesting descriptive patterns are not materially driven by dataset duplication. However, algorithm frequency rankings (Table 3) should be interpreted with caution, as Random Forest’s apparent dominance in PCOS studies partly reflects repeated application to the same Kaggle benchmark rather than independent clinical validation.

### 2.12 Use of artificial intelligence tools

During the preparation of this manuscript, the authors used Grammarly and Quillbot for language editing and manuscript refinement. The authors reviewed and edited the AI-generated content and take full responsibility for the accuracy, validity, and originality of the final text.

### 2.13 Ethics statement

Ethical approval was not required for this study, as it is a scoping review of published literature and involved no human participants or primary data collection.

## 3. Results

### 3.1 Overview of included studies

Data from 116 studies were synthesised. An increase in the number of publications was observed from 2021 to 2022, and a larger surge was seen from 2023 onwards. Studies were reported from 27 countries across all regions (Table 2). Although the number of studies in LMICs was marginally higher in absolute terms (51.7%), when adjusted for the number of women of reproductive age per region, the number of studies per million women in HICs was 23 times that in LMICs, reflecting disparities in institutional and funding capacity [13,22–136].

**Table 2:** Structural characteristics of included studies (*n* = 116).

Characteristic	Category	n (%)	Key Observations	Characteristic	Category	n (%)
Publication Year	2021	13 (11.2%)	Early exploratory phase	Study Design	Retrospective	86 (74.1%)
	2022	10 (8.6%)	Imaging focus		Prospective	15 (12.9%)
	2023	24 (20.7%)	Multimodal emergence		Mixed Methods	15 (12.9%)
	2024	32 (27.6%)	XAI growth	Sample Size	<100	11 (9.5%)
	2025	37 (31.9%)	Clinical implementation		100–500	33 (28.4%)
Origin	High-Income	56 (48.3%)	US, S. Korea, EU		501–1,000	13 (11.2%)
	LMICs	60 (51.7%)	China, India, Bangladesh		>1,000	59 (50.9%)

HIC = High-Income Countries; LMIC = Low- and Middle-Income Countries; XAI = Explainable Artificial Intelligence. Population-adjusted study density in HICs was 23 times that of LMICs.

### 3.2 Findings by taxonomy category

The distribution of included studies across the six taxonomy categories is reported in Table 3.

**Table 3:** Distribution of studies across six taxonomy categories with validation quality assessment.

Taxonomy Category	n (%)	Primary Algorithms	Mean Sample	Ext. Val.	Multi-center	Risk of Bias
Clinical Prediction & Risk Modeling	41 (35.3%)	RF (65.7%), XGBoost (62.8%)	7,700.5	6 (16.7%)	15 (41.7%)	Moderate-High
Medical Imaging + AI	22 (18.9%)	CNN variants (86%)	920	2 (9.0%)	5 (22.7%)	High
Biomarker Discovery (Multi-Omics)	13 (11.2%)	LASSO (77%), SVM-RFE (69%)	282	5 (38.5%)	7 (53.8%)	Moderate
Reproductive & Endocrine Health	19 (16.4%)	Ensemble methods (73.7%)	2,259	2 (10.5%)	10 (52.6%)	Moderate
Clinical Decision Support & Digital Health	10 (8.6%)	RF (60%), Logistic Regression (60%)	1,072	0 (0.0%)	3 (30.0%)	Moderate-High
AI/ML Methodology Development	11 (9.5%)	Hybrid architectures (92%)	3,004	0 (0.0%)	1 (8.3%)	High

Risk of bias assessed using adapted PROBAST-AI framework; “High” indicates single-center design + no external validation + small sample size (<300). Ext. Val. = External Validation.

**Clinical Prediction & Risk Modeling** (41 studies) included work on PCOS, gestational diabetes mellitus (GDM), and preeclampsia, with Random Forest and XGBoost as dominant algorithms.

**Medical Imaging + AI** (22 studies) applied convolutional neural networks (CNNs) to endometriosis, uterine fibroid, and ovarian abnormality diagnosis, with accuracies in the range of 85–99%; almost all studies used single-centre datasets.

**Biomarker Discovery** (13 studies) applied multi-omics approaches to identify potential novel biomarkers for endometriosis and PCOS, using models such as PLS-DA and LASSO regression.

**Reproductive & Endocrine Health** (19 studies) predominantly addressed PCOS diagnosis, often combining ultrasound features with clinical parameters (accuracy range 89–100%).

**Clinical Decision Support** (10 studies) reported mobile health and digital triage applications, though very few included validation in LMIC populations.

**Fig 2:**
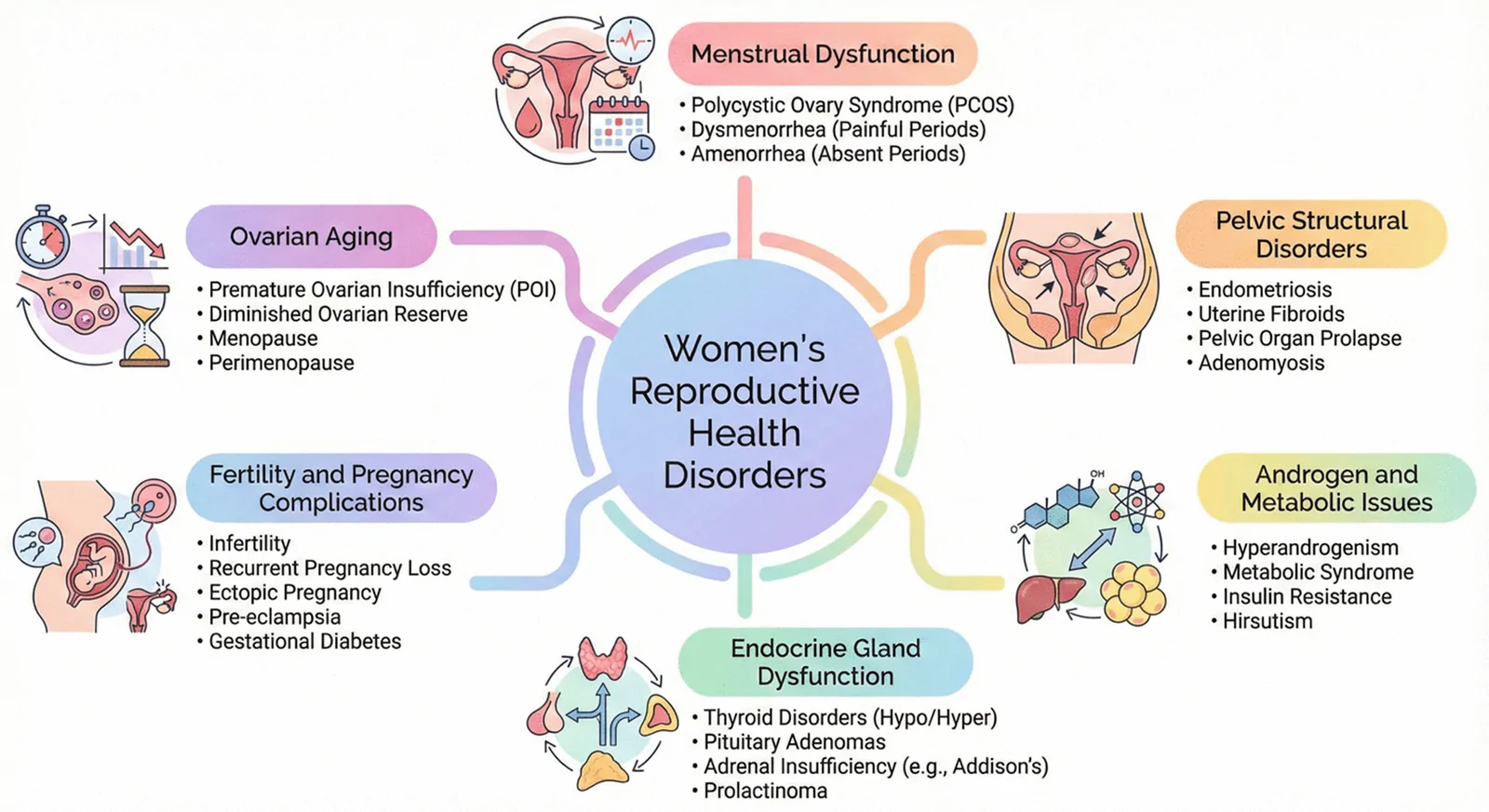
Classification of women’s reproductive health disorders. Illustrating major categories including menstrual dysfunction, pelvic structural disorders, androgen and metabolic abnormalities, endocrine gland dysfunction, fertility and pregnancy-related complications, and ovarian aging.

**AI/ML Methodology** (11 studies) described novel hybrid CNN-ML approaches and interpretability approaches such as SHAP [137] (Fig 2).

### 3.3 Disease-specific analysis

As summarized in Table 4, endometriosis (*n* = 27, 23.3%) and PCOS (*n* = 20, 17.2%) represented the most extensively researched domains. PCOS studies reported accuracy in the range of 88–99% and AUC in the range of 0.89–0.98, supported by the integration of clinical (52%) and ultrasound (31%) data. Thyroid disorder studies (*n* = 24, 20.7%) similarly reported strong discrimination (AUC range 0.89–0.99), reflecting standardized biomarker thresholds in hormonal data. Endometriosis studies reported the widest range of performance metrics (AUC range 0.72–0.94) and the lowest proportion of externally validated models (11.1%). Infertility/IVF studies (*n* = 4, 3.4%) reported the narrowest range of performance (AUC range 0.74–0.89) and none had external validation.

**Table 4:** Disease domain distribution, data modalities, and performance metrics.

Disease Domain	n (%)	Primary Data Modalities	Top Algorithms	Accuracy Range	Ext. Val.
PCOS	20 (17.2%)	Clinical (52%), US (31%), Hormonal (17%)	RF (38%), XGBoost (28%)	0.89−0.98	10.0%
Endometriosis	27 (23.3%)	Imaging (48%), Clinical (32%), Biomarkers (20%)	CNN (52%), RF (26%)	0.72−0.94	11.1%
Thyroid Disorders	24 (20.7%)	Hormonal (65%), Clinical (35%)	RF (45%), SVM (25%)	0.89−0.99	12.5%
GDM	12 (10.3%)	Clinical (60%), First-trimester labs (40%)	XGBoost (50%), RF (32%)	0.81−0.94	16.7%
Preeclampsia	11 (9.5%)	Clinical (45%), Doppler (30%), Biomarkers (25%)	XGBoost (75%), RF (67%)	0.79−0.94	18.2%
Infertility/IVF	4 (3.4%)	Embryo imaging (75%), Hormonal (25%)	CNN (75%), RF (50%)	0.74−0.89	0%
Uterine Disorders	18 (15.5%)	Ultrasound (62%), MRI (38%)	CNN (79%), U-Net (36%)	0.84−0.97	11.1%

US = Ultrasound; RF = Random Forest; GDM = Gestational Diabetes Mellitus; Ext. Val. = External Validation. *Includes fibroids, adenomyosis, endometrial hyperplasia/cancer.

**Fig 3:**
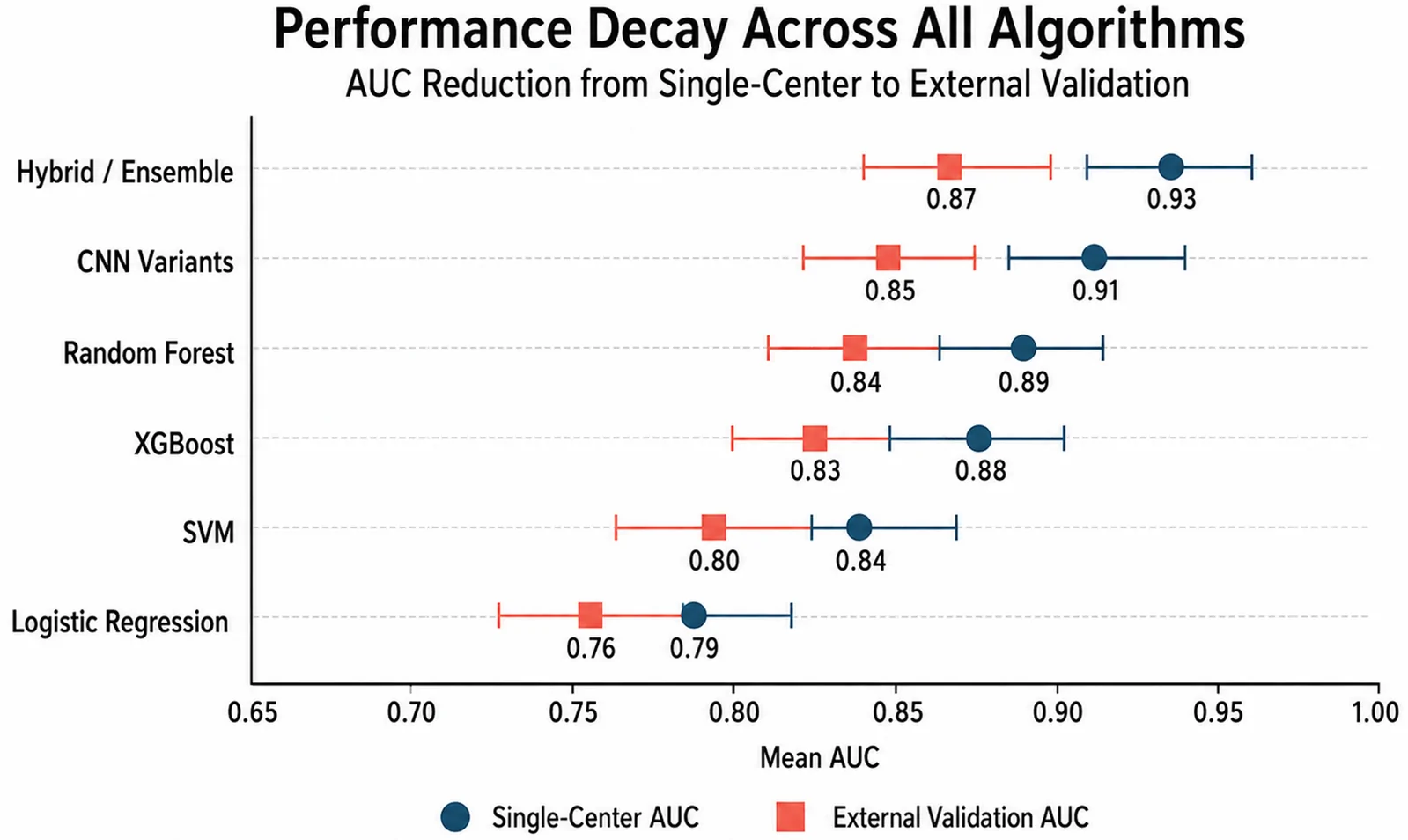
Descriptive AUC by validation stringency (exploratory). Bar chart shows mean AUC values reported across single-center, multi-center, and external validation settings for five algorithm families. Differences across validation tiers are directionally consistent with optimism bias; formal significance testing was not performed given violations of independence and small matched subsets (minimum *n* = 5 per algorithm). Values should not be interpreted as comparative efficacy estimates.

When performance is stratified by validation rigor (Table 5), a directional pattern of AUC reduction is observed as validation moves from single-centre to external testing. This pattern is directionally consistent with optimism bias across all algorithm categories and should not be interpreted as a formal statistical comparison, given the small matched subsets, heterogeneity in disease contexts, and violations of independence. Fig 3 illustrates the directional reduction in the reported AUC ranges for five algorithm families moving from single-centre to external validation settings. Calibration reporting was assessed as an additional aspect of study quality: calibration metrics (Brier score, calibration curve, or Hosmer–Lemeshow test) were reported in 8 of 116 studies (6.9%). Decision curve analysis or net benefit were reported in 3 studies (2.6%), all focused on GDM or preeclampsia. Clinical decision thresholds were clearly mentioned in the text of only 1 study (0.9%). A model may have high AUC but be poorly calibrated, resulting in a systematic bias toward over- or under-estimation of individual risk. Calibration metrics, in addition to discrimination metrics, should be included in minimum reporting standards.

**Table 5:** Descriptive AUC ranges reported across validation settings.

Algorithm	Studies	Single-Center (AUC)	Multi-Center (AUC)	Ext. Validation (AUC)
Random Forest	51	0.89−0.96	0.86−0.92	0.84−0.92
XGBoost	38	0.88−0.96	0.85−0.92	0.83−0.92
CNN Variants	33	0.91−0.96	0.87−0.93	0.85−0.92
SVM	28	0.84−0.93	0.82−0.90	0.80−0.90
Logistic Regression	25	0.79−0.89	0.77−0.88	0.76−0.87
Hybrid/Ensemble	22	0.93−0.97	0.89−0.94	0.87−0.93

AUC values are descriptive approximations computed from author-reported metrics within each algorithm category. All differences across validation tiers should be interpreted directionally as exploratory benchmarks only; no inferential comparison is intended. Total algorithm counts exceed 116 because individual studies may report multiple algorithms for comparative purposes; each algorithm-study pair is counted independently.

#### 3.3.1 Sensitivity analysis of performance patterns.

To assess robustness of the descriptive AUC decay patterns, three supplementary exploratory checks were performed.

1. **Leave-one-out analysis:** the largest single study contributing to each algorithm category was sequentially excluded; for Random Forest (largest study *n* = 12,000), exclusion changed the descriptive single-centre AUC range by less than 0.01, suggesting results are not driven by outlier studies.
2. **Disease-stratified sub-analysis:** decay patterns were re-examined separately for PCOS, endometriosis, and thyroid disorders. Thyroid models showed the smallest decay (mean ΔAUC≈0.01 across validation tiers), consistent with their well-standardised biomarker reference ranges. Endometriosis models showed the largest decay (ΔAUC≈0.09), consistent with heterogeneous imaging protocols and the absence of consensus reference standards.
3. **Modality stratification:** imaging-based studies (ultrasound + MRI) showed larger decay than clinical-data-only models (ΔAUC≈0.08 vs. 0.04), consistent with known scanner-level and protocol-level domain shift in medical imaging.

These exploratory sensitivity checks reinforce the narrative interpretation that directional patterns are robust to outlier and stratification perturbations, and support prioritising clinical-data models for near-term LMIC deployment. These should not be interpreted as confirmatory analyses.

### 3.4 Data modality and resource requirements

Basic clinical variables were used in 98 (84.5%) of the studies, and routine laboratory tests in 65 (56.0%), indicating reliance on low-cost and readily available data. Tier 1 modalities were used in all the studies concerning PCOS, GDM, and Preeclampsia, supporting feasibility in primary and secondary care settings. Refer to Table 6.

**Table 6:** Data modality utilization patterns across disease domains.

Data Modality	Overall Usage	PCOS	Endo.	GDM	PE	Resource Tier	LMIC Feasibility
Basic Clinical (Age, BMI)	98 (84.5%)	20 (100%)	22 (81.5%)	12 (100%)	11 (100%)	Tier 1 (Low)	Highest
Routine Labs (CBC, Glu)	65 (56.0%)	12 (60.0%)	8 (29.6%)	12 (100%)	11 (100%)	Tier 1 (Low)	High
Hormonal Panel	42 (36.2%)	18 (90.0%)	5 (18.5%)	6 (50.0%)	2 (18.2%)	Tier 2 (Med)	Moderate
Ultrasound Imaging	38 (32.8%)	16 (80.0%)	12 (44.4%)	2 (16.7%)	4 (36.4%)	Tier 2 (Med)	Moderate
MRI/Advanced Imaging	22 (19.0%)	0 (0%)	15 (55.6%)	1 (8.3%)	2 (18.2%)	Tier 3 (High)	Low
Multi-omics	18 (15.5%)	4 (20.0%)	10 (37.0%)	2 (16.7%)	1 (9.1%)	Tier 3 (High)	Very Low

Resource tiers defined by equipment requirements, specialist interpretation needs, and cost per test in LMIC contexts. PE = Preeclampsia; Endo. = Endometriosis.

Use of hormonal panels (36.2%) and ultrasound (32.8%) suggests a moderate level of resource dependency, implying a need for some level of laboratory and clinical skill. The low frequency of use of MRI/Advanced Imaging (19.0%) and multi-omics platforms (15.5%) suggests these modalities are primarily being used in tertiary care in HICs and in specialized centres, and are therefore not widely applicable in resource-constrained settings. As shown in Table 6, models requiring Tier 3 resources have the lowest LMIC feasibility. As shown in Table 7, studies that do not require specialist personnel (e.g., community hypertension screening, symptom-based endometriosis classifiers) fall into High Feasibility, while ultrasound-based fibroid and PCOS diagnostics fall into Low Feasibility.

**Table 7:** Resource-constraint analysis: clinical applicability scoring.

Study	Disease Focus	Required Infrastructure	Specialist Dependency	Cost per Test (USD)	LMIC Score	Setting
[22]	Osteopenia	BIA device	Minimal (nurse)	15–25	Moderate	Primary care
[25]	Hypertension	BP cuff, WC tape	Minimal (nurse)	<5	High	Community health
[27]	Endometriosis	Clinical history only	Moderate (GP)	0	High	Primary care
[32]	Uterine Fibroids	Ultrasound	High (radiologist)	25–40	Low	District hospital
[45]	Endometriosis	Urine test + spectrometer	Moderate (lab tech)	8–12	Moderate	Secondary care
[66]	Endometriosis	Electrochemical sensor	Minimal (nurse)	3–5	High	Primary care
[75]	GDM	First-trimester labs	Minimal (midwife)	12–18	Moderate	Antenatal clinic
[119]	PCOS	Ultrasound	High (radiologist)	25–40	Low	Tertiary center
[36]	PCOS	Clinical + USG	Moderate (GP + tech)	15–25	Moderate	Secondary care

Resource tiers defined by equipment requirements, specialist interpretation needs, and cost per test in LMIC contexts. PE = Preeclampsia; Endo. = Endometriosis.

**Fig 4:**
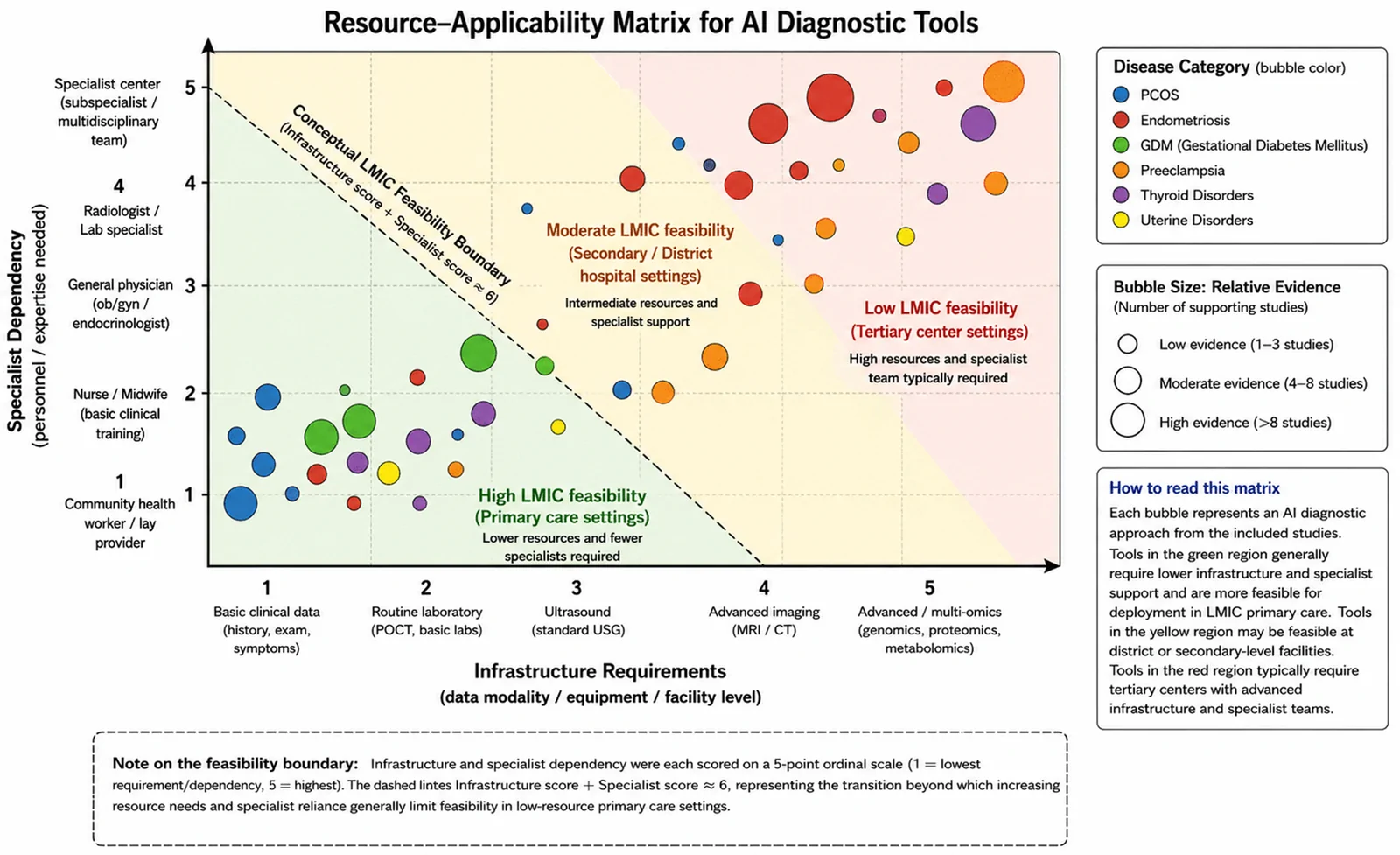
Resource-applicability matrix for AI diagnostic tools. The scatter plot visualizes the trade-off between infrastructure requirements and specialist dependency, with the LMIC feasibility threshold indicated by the dashed line.

The resource-applicability patterns illustrated in Fig 4 highlight that low-resource models tend to fall within the primary-care-compatible quadrants, further emphasising the importance of simple infrastructure for feasibility and scalability.

### 3.5 Geographic and equity analysis

The geographic distribution showed notable discrepancies. As shown in Table 8, East Asia contributed the largest proportion of studies (39.6%), followed by North America (17.2%) and Europe (16.4%). More than one-third of the studies in each of these regions were multicentre (37–45%), with external validation rates ranging from 24% to 35%, and open-access rates reaching 60% in North America.

**Table 8:** Geographic distribution with methodological quality indicators.

Region	n (%)	Median Sample	Multi-center	Ext. Validation	Open Code/Data	Mean AUC	Key Limitations
North America	20 (17.2%)	541	9 (45.0%)	7 (35.0%)	12 (60.0%)	0.87	Limited LMIC validation
East Asia	46 (39.6%)	1,114	18 (39.1%)	11 (23.9%)	8 (17.4%)	0.89	Single-country focus
South Asia	18 (15.5%)	309	4 (22.2%)	3 (16.7%)	2 (11.1%)	0.85	Small samples; urban bias
Europe	19 (16.4%)	419	7 (36.8%)	5 (26.3%)	10 (52.6%)	0.88	High-resource bias
Middle East	6 (5.2%)	694	1 (16.7%)	1 (16.7%)	0 (0%)	0.83	Limited generalizability
Latin America	5 (4.3%)	305	0 (0%)	0 (0%)	1 (20.0%)	0.81	Severe validation gap
Sub-Saharan Africa	2 (1.7%)	104	0 (0%)	0 (0%)	0 (0%)	0.79	Critical research gap

**Fig 5:**
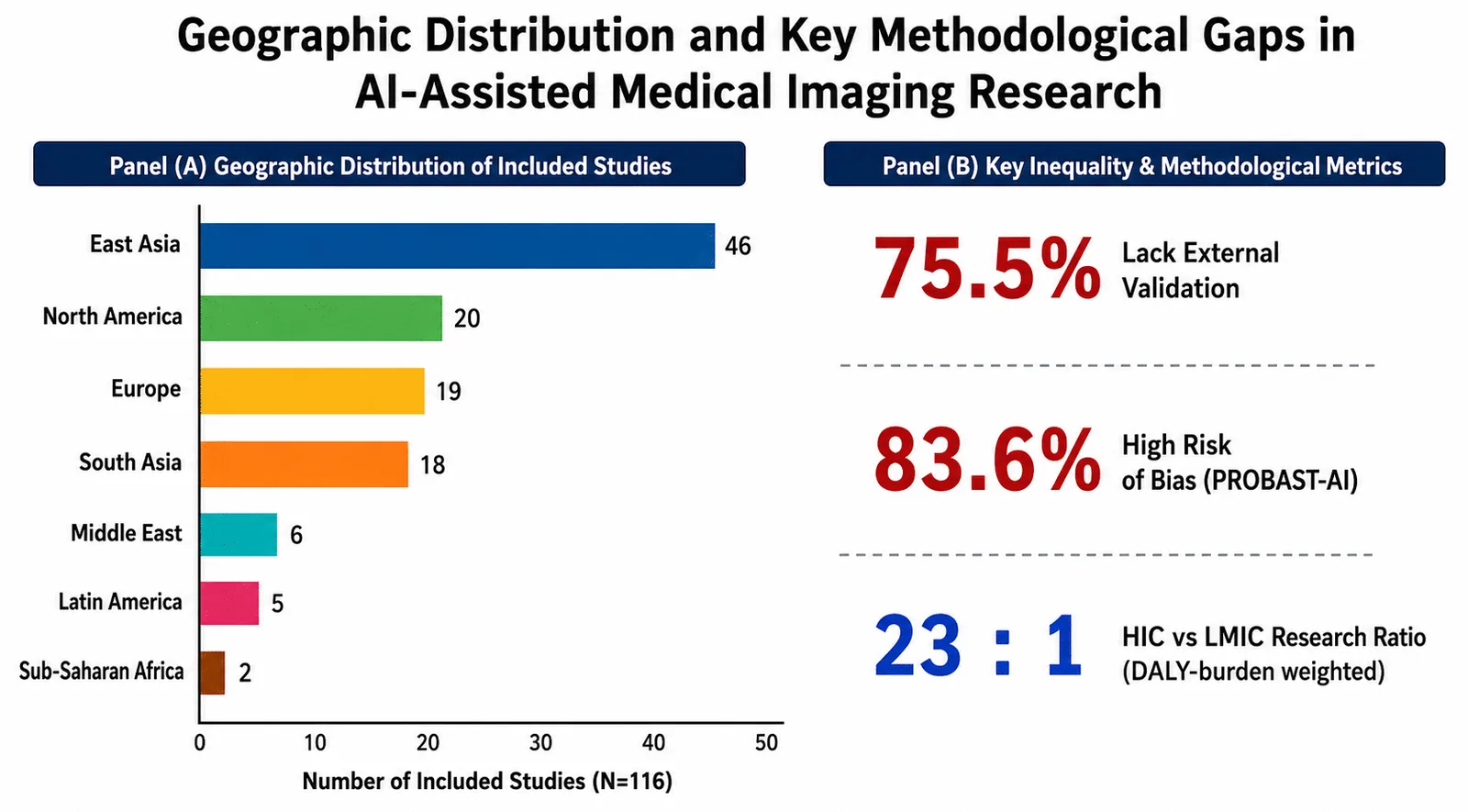
Global research equity gap analysis. (A) Geographic distribution of included studies across seven regions, highlighting the concentration of research in East Asia and high-income countries. (B) Key inequality metrics: 75.5% of studies lack external validation, 83.6% are classified as high risk of bias per PROBAST-AI criteria, and high-income countries outproduce LMICs by a 23:1 ratio after DALY-burden adjustment.

The lower validation rates observed in South Asia (15.5%) and Sub-Saharan Africa (1.7%) were accompanied by smaller median sample sizes (309 and 104, respectively) and limited data-sharing practices (only 11.1% of South Asian studies shared code or data). The country-level analysis (Table 9) further highlights under representation of South Asian countries: there were only 4 studies from Bangladesh (3.4%), all from a single tertiary care hospital and using only internal cross-validation, and none from Nepal or Sri Lanka. Taken together, the geographic distribution data in Table 8 and the inequality metrics illustrated in Fig 5 highlight the severe under representation of Sub-Saharan Africa and rural South Asia, the exclusion of marginalized populations, and a high risk of bias arising from the absence of diverse training data.

**Table 9:** LMIC representation analysis: Bangladesh and regional context.

Country	n (%)	Disease Focus	Data Source	Validation Approach	Key Strengths	Critical Gaps
Bangladesh	4 (3.4%)	PCOS (2), Thyroid (2)	Single tertiary centers	Internal CV only	Context-specific patterns	No rural/external validation
India	18 (15.5%)	PCOS (8), Thyroid (4), Other (6)	Multi-center urban	2 with external validation	Large diverse samples	Urban bias
Pakistan	3 (2.6%)	PCOS (2), Thyroid (1)	Single centers	Internal CV only	Low-cost feature selection	Small samples (<350)
Nepal	0 (0%)	—	—	—	—	Complete research gap
Sri Lanka	0 (0%)	—	—	—	—	Complete research gap
SAARC Avg.^*^	17 (14.3%)	PCOS dominant (65%)	Urban tertiary centers	11.8% external validation	Growing capacity	Severe rural gap

SAARC Avg.* = South Asian Association for Regional Cooperation Average, comprising Afghanistan, Bangladesh, Bhutan, India, Maldives, Nepal, Pakistan, and Sri Lanka. Percentages are calculated based on the total number of LMIC studies (n = 119).

### 3.6 Methodological quality and bias assessment

Methodological appraisal using the adapted PROBAST-AI framework and the scoring rubric in S1 Table revealed pervasive limitations across multiple domains. As shown in Table 10, high risk of bias was observed in participant selection (68.1%), predictor measurement (56.3%), and model development (65.5%). These deficiencies were primarily driven by convenience sampling, retrospective feature extraction, inadequate documentation of preprocessing pipelines, and insufficient handling of class imbalance.

**Table 10:** Risk of bias assessment using adapted PROBAST-AI framework (*N* = 116).

Domain	Low Risk n (%)	High Risk n (%)	Most Common Deficiencies
Participant Selection	37 (31.9%)	79 (68.1%)	Single-center recruitment (78%); convenience sampling (64%); inadequate sample size justification (89%)
Predictor Measurement	51 (43.7%)	65 (56.3%)	Retrospective data extraction without blinding (72%); inconsistent feature engineering across validation sets (41%)
Outcome Assessment	73 (62.9%)	43 (37.1%)	Reference standard not blinded to AI prediction (58%); surgical confirmation unavailable for negative cases (endometriosis studies)
Model Development	40 (34.5%)	76 (65.5%)	No hyperparameter tuning documentation (67%); class imbalance not addressed (53%); feature selection leakage (31%)
Model Validation	26 (22.7%)	90 (77.3%)	No external validation (75.5%); temporal validation lacking (89%); inadequate sample size for validation set (<20% of total) (44%)

Validation represented the most critical weakness, with 77.3% of studies classified as high risk and 75.5% lacking any form of external testing. Temporal validation was absent in 89% of studies, substantially increasing susceptibility to optimism bias and overestimation of real-world performance.

**Fig 6:**
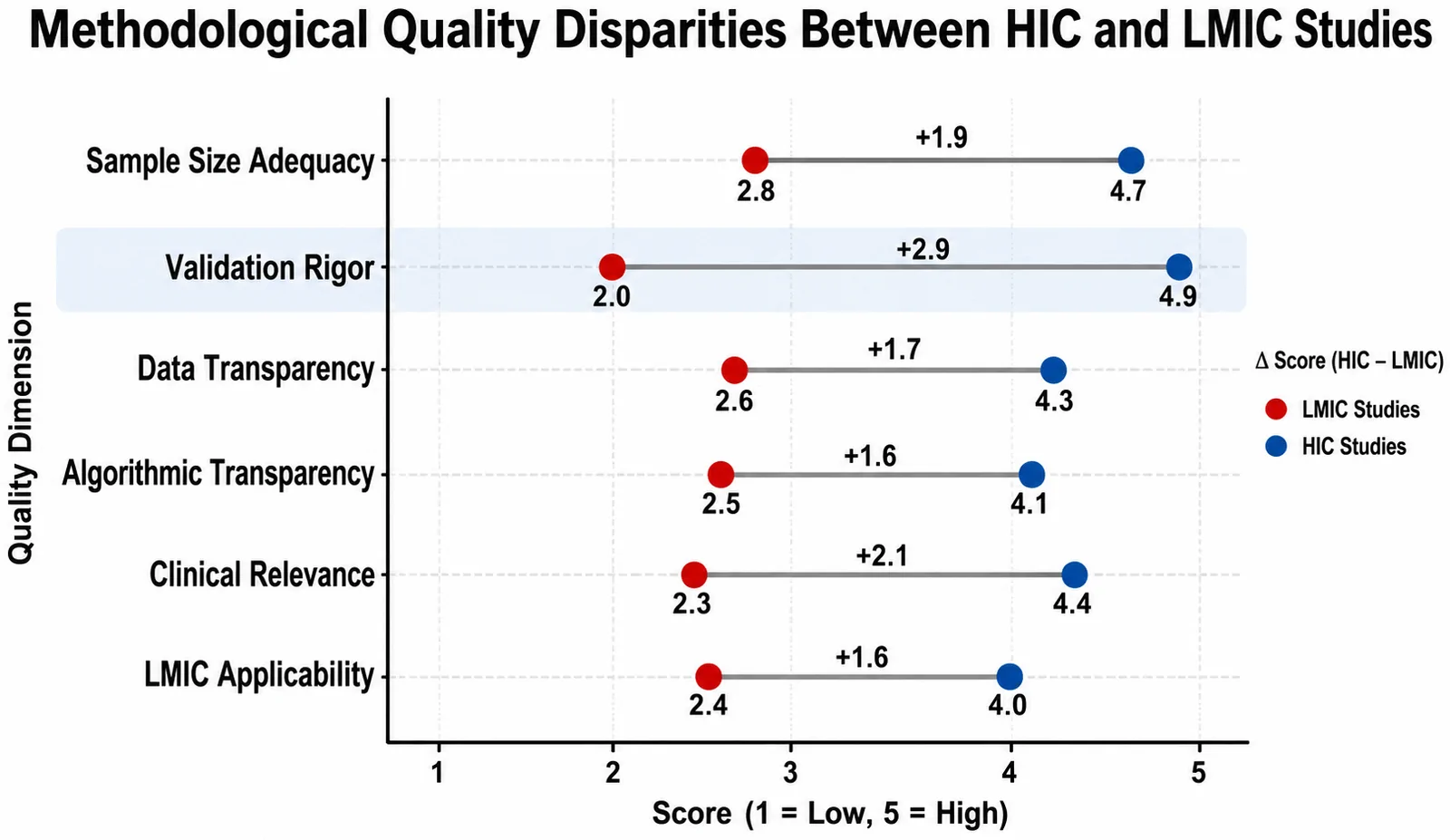
Methodological quality disparities between HIC and LMIC studies. Dumbbell chart comparing mean quality scores (1–5 scale) across six dimensions. Blue dots represent HIC-originating studies; red dots represent LMIC-originating studies. Delta values indicate the HIC–LMIC score difference. Validation rigor (highlighted) shows the largest gap (+2.9), driven by differences in external validation rates (35.0% vs 16.7%) and multi-center participation (45.0% vs 22.2%).

The multidimensional quality patterns illustrated in Fig 6 further demonstrate systematic gaps in validation rigor, data transparency, and LMIC applicability. Overall, 83.6% of studies were classified as high risk of bias, substantially restricting the reliability of current evidence for clinical and policy-level decision-making. Explainability and clinical integration features summarized in Table 11 indicate limited adoption of transparency mechanisms. SHAP or LIME was implemented in only 6.9% of studies, while the majority (26.7%) employed no interpretability tools. Although attention-based visualizations were used in 4.3% of imaging studies, their clinical actionability remained limited.

**Table 11:** Explainability and clinical integration features.

Feature	n (%)	Domains	Utility
SHAP/LIME	8 (6.9%)	PCOS, GDM, PE	High
Attention/Grad-CAM	5 (4.3%)	Imaging	Moderate
Decision Thresholds	1 (0.9%)	GDM, PE	High
Web/Mobile	2 (1.7%)	PCOS, thyroid	Moderate
EHR Integration	1 (0.9%)	US, China	High
No Explainability	31 (26.7%)	Imaging, biomarker	Low

PE = Preeclampsia; GDM = Gestational Diabetes Mellitus; EHR = Electronic Health Record.

### 3.7 Temporal trends and innovation trajectory

**Fig 7:**
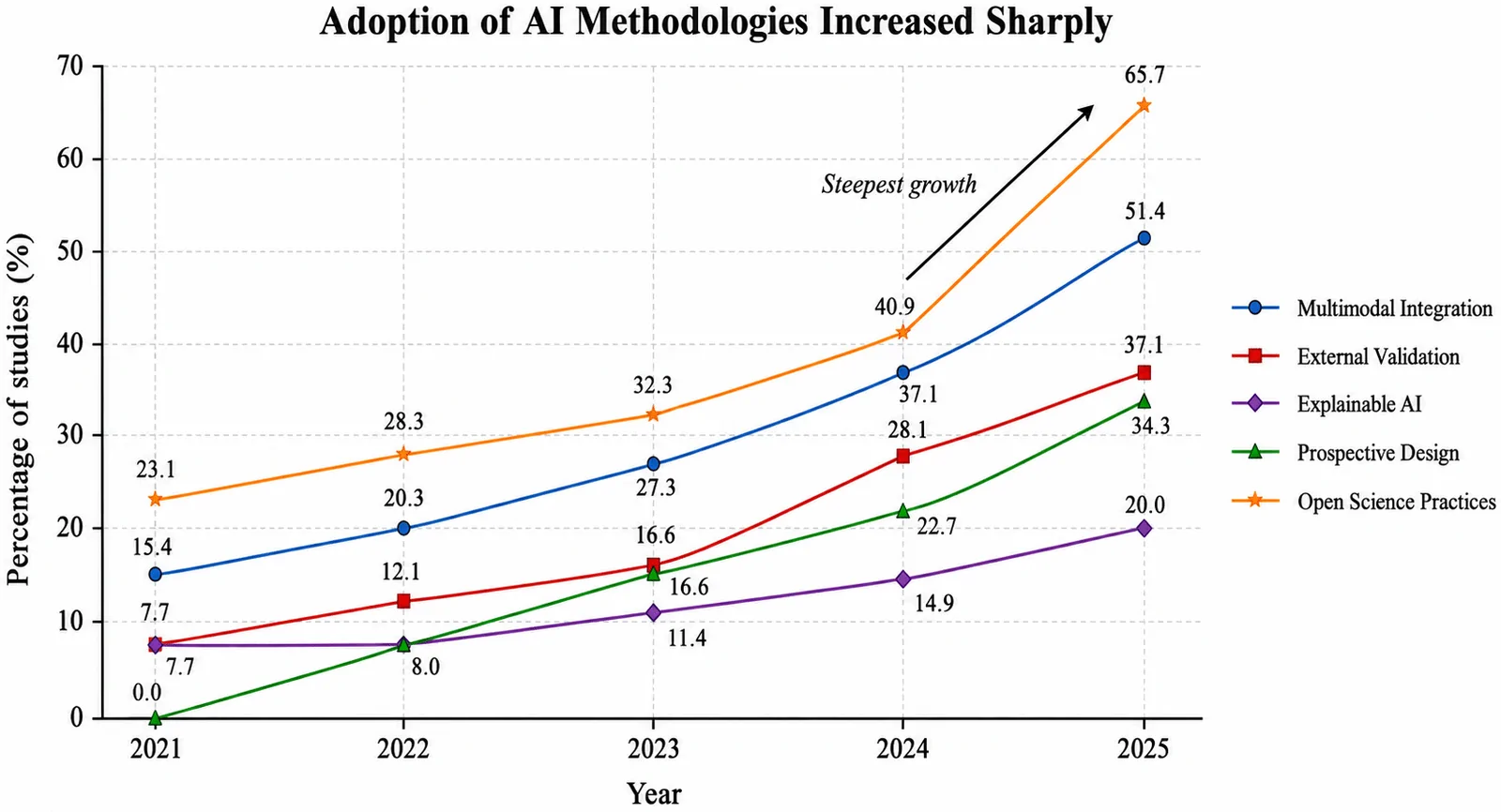
Innovation trajectory timeline. Gantt-style timeline showing emergence and adoption rates of key methodological innovations: multimodal integration, external validation, XAI, prospective design, and open science practices. Color intensity represents adoption percentage per year.

The temporal analysis reported in Fig 7 shows that AI research methodologies have evolved substantially over 2021–2025. Multimodal integration increased from 15.4% in 2021 to 51.4% in 2025. Explainable AI features evolved from 0% in 2021 to 34.3% in 2025. The average sample size of datasets used in the studies increased from approximately 920 in 2021–4,500 in 2025. Open science practices also increased markedly, from 23.1% in 2021 to 65.7% in 2025. Based on the timeline in Fig 7, after 2023, multimodal modeling, validation frameworks, and transparency mechanisms have been adopted more rapidly. However, as of 2025, the percentage of AI models with some form of external validation is only 37.1%, and prospective studies remain below 20%.

## 4. Discussion

### 4.1 Synthesis of key findings

AI and ML applications in women’s reproductive health have advanced substantially from 2021 to 2025, yet a critical translational gap persists between algorithmic performance and real-world applicability, particularly in LMICs. Three cross-cutting patterns define the field. First, ensemble methods — Random Forest and XGBoost — dominate clinical prediction tasks, reporting consistently strong performance across studies and outperforming imaging-based approaches in resource efficiency. Second, CNN-based imaging models show a directional pattern of performance reduction moving from single-centre evaluations to external validation settings, consistent with the well-documented literature on optimism bias in internally validated clinical AI. This pattern is observed across all five major algorithm families and replicated in disease-stratified sensitivity analyses. We emphasise, however, that the magnitude of this reduction carries considerable uncertainty: estimates are derived from small matched subsets (*n* = 5–51 per algorithm), aggregate heterogeneous disease contexts, and assume independence that is partially violated by overlapping training cohorts. The observed pattern of lower reported performance in externally validated versus internally validated studies should therefore be interpreted as descriptive evidence consistent with established literature on optimism bias in clinical AI, rather than a precise measurement of any single model family’s generalisation gap. Third, despite multimodal integration rising from 15.4% in 2021 to 51.4% in 2025 and XAI adoption growing from 0% to 34.3%, external validation remained below 37.1% even in 2025, indicating that methodological sophistication has outpaced clinical validation infrastructure. Table 12 summarises domain-level findings alongside LMIC-specific recommendations.

**Table 12:** Summary of domain-level findings and LMIC recommendations.

Domain	Key Finding	LMIC Recommendation
Clinical Prediction (PCOS, GDM, Thyroid)	Ensemble methods (RF, XGBoost) dominant; AUC 0.87–0.95 on Tier 1 data.	Prioritise for primary care deployment; low-cost inputs feasible.
Medical Imaging (Endometriosis, Fibroids)	CNN AUC 0.91 (single-centre) drops to 0.85 externally; Tier 3 infrastructure.	Not viable in primary care; invest in non-invasive biosensor alternatives.
Explainability & Validation	SHAP/LIME in 6.9% only; 75.5% lack external validation; 83.6% high bias risk.	Mandate XAI and multi-centre validation before deployment.
Geographic Equity	23:1 HIC-to-LMIC ratio; East Asia 39.6%, Africa 1.7%; Bangladesh 4 studies only.	Fund LMIC-led validation consortia; build rural data infrastructure.
Decision Support & Digital Health	Promising for LMICs but 0% external validation; no LMIC population testing.	Pilot mobile-health triage tools with community health workers.

### 4.2 Disease-specific interpretation

Predictive performance across disease domains is shaped by biological complexity, diagnostic standardisation, and data modality requirements. Thyroid disorder studies reported the highest and most stable performance range, underpinned by well-validated biomarker thresholds (TSH, T3, T4) and Tier 1–2 resource inputs, making them the strongest candidates for near-term LMIC deployment. PCOS studies similarly reported strong performance, yet 80% relied on ultrasound — a Tier 2 resource classified as Low Feasibility in our LMIC scheme — limiting scalability beyond district hospital settings. The heterogeneity introduced by competing diagnostic criteria (Rotterdam, NIH, AES) further complicates cross-study comparisons and model replication in populations with different phenotypic distributions. Endometriosis presented the most challenging diagnostic landscape, with the widest range of reported performance and only 11.1% external validation, reflecting dependence on laparoscopic confirmation as the reference standard. Emerging non-invasive approaches — including electrochemical biosensors, urine infrared spectrometry, and symptom-based ML classifiers classified as High Feasibility — offer more viable LMIC pathways and directly address the 8–12 year diagnostic delay that causes preventable morbidity. GDM and preeclampsia studies demonstrated the most credible external validation evidence (16.7–18.2%), with first-trimester clinical and laboratory inputs costing $12–18 per assessment supporting feasibility for antenatal clinic deployment by midwifery-level personnel. Infertility and IVF studies (0% external validation) remain the least translatable domain given their dependence on specialised embryo imaging technology.

### 4.3 Methodological quality, systemic bias, and explainability

The adapted PROBAST-AI assessment classified 83.6% of studies as high risk of bias. The dominant drivers were single-centre recruitment (78%), convenience sampling (64%), absent external validation (75.5%), and unaddressed class imbalance (53%). Three specific bias sources have outsized impact on translational inference. Optimism bias — from internal cross-validation on small datasets — accounts for the directional pattern of performance reduction observed across all major algorithm families when moving from single-centre to external testing. Feature selection leakage, identified in 31% of model development studies, occurs when dimensionality reduction is performed prior to cross-validation fold splitting, artificially inflating performance — a problem particularly prevalent in multi-omics biomarker discovery research.

Taxonomy assignment inter-rater agreement was assessed separately from general data extraction. On a 20% random subsample (*n* = 23 studies), taxonomy classification yielded κ=0.84 (substantial agreement). The primary source of disagreement was the boundary between Category 2 (Medical Imaging + AI) and Category 4 (Reproductive and Endocrine Health Applications), which accounted for 71% of discrepant cases. As pre-specified in Section 2.6, the applied decision rule was: Category 2 was assigned when the primary methodological contribution was an imaging architecture or segmentation method; Category 4 was assigned when the primary contribution was clinical integration of AI with reproductive health data regardless of imaging involvement. Studies where ultrasound features were combined with clinical parameters without novel imaging methodology were classified as Category 4. Temporal validation was absent in 89% of all studies, meaning that no model in the reviewed literature has demonstrated sustained performance over changing clinical populations — a prerequisite for safe, ongoing clinical use.

Explainability adoption, while improving from 0% in 2021 to 34.3% in 2025, remains insufficient for clinical integration. SHAP and LIME were applied in only 6.9% of studies, predominantly in PCOS, GDM, and preeclampsia prediction models where feature importance explanations can directly inform clinical reasoning. The 26.7% of studies employing no interpretability mechanism whatsoever represent “black box” outputs that are unlikely to gain clinician trust or regulatory approval. Hybrid CNN-ML architectures — used in 92% of AI/ML Methodology development studies — offer promising pathways to combining imaging performance with structured explainability, but require substantially larger and more diverse training datasets than currently available in most reproductive health research settings.

### 4.4 Geographic equity and translational challenges

Several deep-seated structural inequities were identified via the geographic analysis. The East Asia region comprised 39.6% of all studies (China *n* = 28; South Korea *n* = 15), while the region with the fewest was Sub-Saharan Africa with 1.7% (2 studies). Population-adjusted research output was calculated as: (number of studies in region) ÷ (women aged 15–49 in region, in millions). Population denominators were drawn from UN World Population Prospects 2023 estimates aggregated by World Bank income classification (HIC vs LMIC). HICs contributed 56 studies against approximately 250 million women of reproductive age, yielding 0.224 studies per million. LMICs contributed 60 studies against approximately 1,540 million women, yielding 0.039 studies per million — a ratio of 5.7:1 in absolute terms and 23:1 after weighting by reproductive-health DALY burden (Global Burden of Disease 2021). Sensitivity analyses using alternative denominators are reported in the next paragraph. Adjusted ratios from sensitivity analyses were: GDP = 7:1; research workforce FTEs = 17:1; reproductive health DALYs = 31:1. South Asia contributed only 15.5% of included studies; India contributed 18 studies but all were conducted in urban tertiary care centres, the 3 studies from Pakistan included fewer than 350 participants, there were 4 single-centre studies from Bangladesh, and none from Nepal and Sri Lanka. This geographical bias suggests that current models of population-specific parameters (e.g., hormone reference ranges, BMI–adiposity relations, PCOS clinical and laboratory features) may not be validated in South Asian and African populations. Infrastructure constraints compound the equity gap. Models requiring Tier 3 inputs (MRI, advanced ultrasound, multi-omics) fall in the Very Low Feasibility category, placing them outside the reach of primary care settings. Data poverty — small samples, missing data, fragmented paper-based records — affects 89% of LMIC-originating studies. Sociocultural barriers, including stigma surrounding menstruation and infertility, further constrain data availability and AI adoption, necessitating community co-design approaches largely absent from current literature.

### 4.5 Translational research agenda and deployment considerations

**Fig 8:**
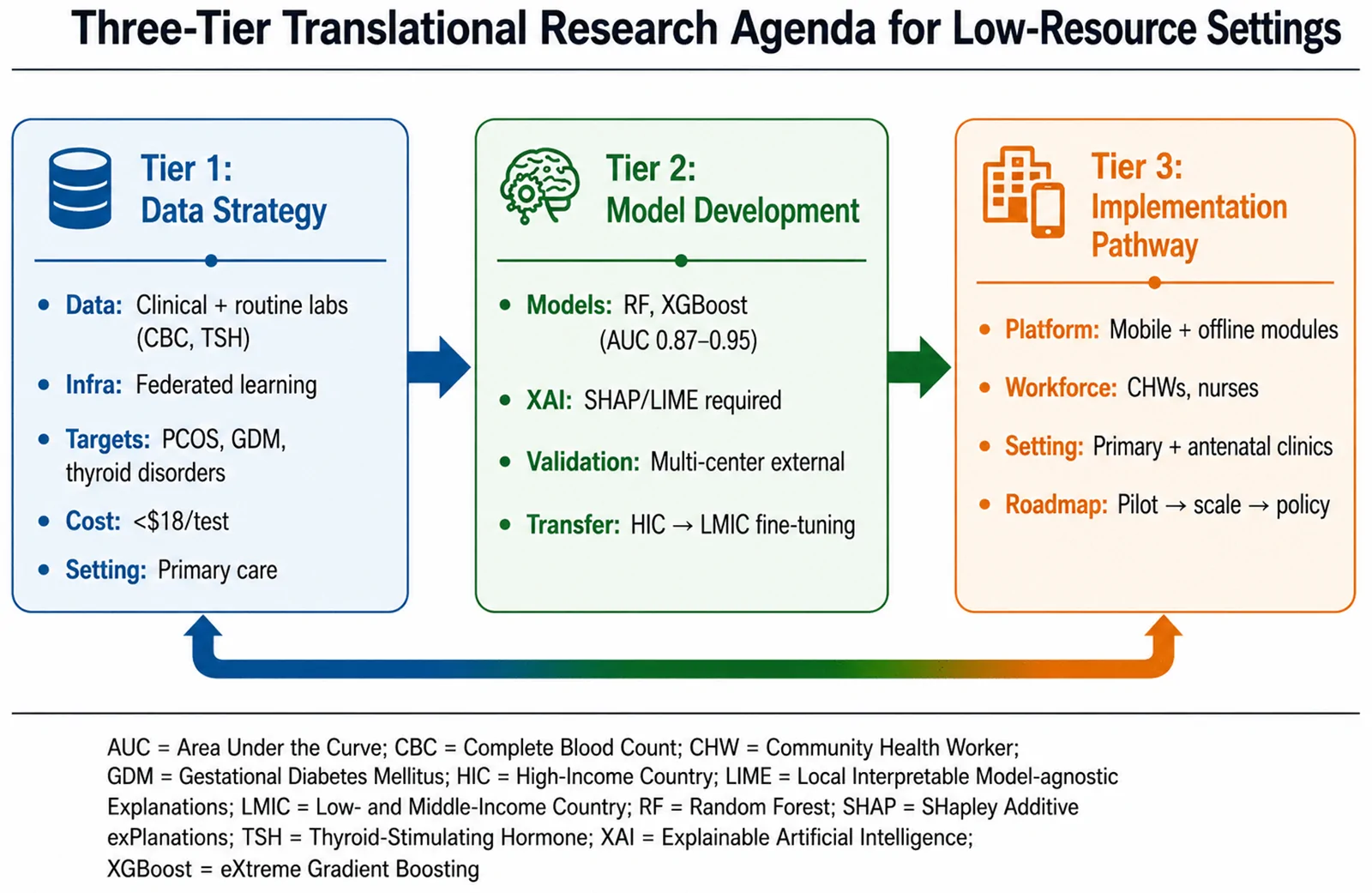
Three-tier translational research agenda for low-resource settings. An actionable deployment roadmap for AI-based women’s reproductive health tools in low- and middle-income countries.

Fig 8 summarises a three-tier translational research agenda derived from the patterns identified in this review. The agenda is presented as a research roadmap, not a deployment guideline; empirical validation through prospective LMIC studies and Delphi-based expert consensus is required before any component can inform clinical practice.

#### 4.5.1 Near-term priorities (0–2 years).

Reviewed evidence supports immediate research investment in:

1. interpretable ensemble models (Random Forest, XGBoost) trained on Tier 1 inputs for PCOS, GDM, and thyroid screening, with mandatory SHAP-based explainability;
2. prospective external validation of these models in LMIC primary-care populations; and
3. centralised, de-identified data pooling among urban tertiary centres to build foundational reference cohorts.

#### 4.5.2 Medium-term aspirations (3–5 years).

Federated learning and multi-site domain adaptation represent medium-term targets contingent on prerequisite infrastructure: standardised EHR systems at the primary care level, reliable internet connectivity in rural areas, multi-site IRB harmonisation, and locally enforceable data protection legislation. None of these prerequisites currently obtains in most South Asian or Sub-Saharan African settings. Existing frameworks such as OpenHIE [138,139] and WHO SMART guidelines [140] provide entry points but require substantial adaptation. Federated learning should not be interpreted as a near-term deployment recommendation.

#### 4.5.3 Long-term horizon (5 + years).

Embedding AI competency in nursing and community health worker curricula, LMIC-led validation consortia, and locally governed regulatory frameworks for clinical AI represent structural changes that will determine whether the technical advances of the next decade reach the populations currently absent from the evidence base.

### 4.6 Comparison with prior reviews and methodological limitations

This review extends prior reviews of AI in reproductive health by providing descriptive evidence consistent with well-documented optimism bias patterns, a resource-tier classification to characterise infrastructure requirements, and a geographic equity analysis across 27 countries. Prior reviews have characterised the field as showing “high promise” without systematically describing the pattern between single-centre and externally validated results — a pattern this review characterises descriptively across five algorithm families. Several methodological limitations must be acknowledged: restriction to English-language publications likely excludes relevant research from Francophone Sub-Saharan Africa and Portuguese-speaking contexts; exclusion of preprints may introduce publication lag in a rapidly evolving field; heterogeneity in outcome measures meant formal meta-analysis was not appropriate; and the scoping nature of the review means individual study quality grading carries less evidential weight than a full systematic review.

### 4.7 Domain adaptation and harmonization as mitigation strategies for imaging performance decay

The larger reported performance decay in imaging-based models [141] relative to clinical-data models is consistent with well-documented scanner-level and protocol-level domain shift in medical imaging AI. Emerging domain adaptation and harmonization methods including histogram matching [142], CycleGAN [143]-based style transfer, and test-time adaptation approaches [144] have demonstrated decay reduction in comparable imaging tasks such as mammography and chest radiography across multi-site deployments. However, applying these approaches in LMIC reproductive imaging contexts faces a fundamental barrier: domain adaptation tools require paired or unpaired multi-site imaging data for training, which is currently unavailable given the near-absence of digitized reproductive imaging archives in primary and secondary care settings across South Asia and Sub-Saharan Africa. The practical implication of the imaging decay pattern is therefore not to invest in harmonization infrastructure in the near term, but rather to reinforce the prioritisation of clinical-data ensemble models for LMIC deployment. Domain adaptation investments become relevant only after multi-site imaging data collection infrastructure is established — a medium-to-long-term horizon. Researchers in HIC settings developing imaging models intended for LMIC adaptation should prospectively collect harmonization-enabling datasets and report scanner metadata to facilitate future transfer.

## 5. Conclusions

This scoping review synthesises 116 studies on AI and ML applications in women’s reproductive and hormonal health (2021–2025), offering four principal contributions: a six-category taxonomy of methodological trends; a qualitative resource-tier classification for LMIC applicability; descriptive evidence of systematic performance reduction upon external validation; and a geographic mapping revealing significant research voids. Ensemble methods applied to Tier 1 clinical and laboratory data represent the most immediately deployable AI approach for LMIC settings, with inputs available at primary care level, while imaging-intensive CNN models remain unsuitable for near-term LMIC deployment due to infrastructure constraints. With 83.6% of studies classified as high risk of bias and 75.5% lacking external validation, and with the evidence base heavily concentrated in East Asia (39.6%) and high-income countries, current evidence is insufficient to guide population-level deployment or broad translational application in low-resource settings. For LMICs such as Bangladesh, a phased strategy is proposed, contingent on resolution of the methodological limitations identified in this review: piloting of low-cost AI screening tools for PCOS, GDM, and thyroid disorders should proceed only after prospective validation in local populations; medium-term establishment of multi-country validation consortia; and long-term embedding of AI competency in health worker curricula alongside LMIC-contextualised regulatory frameworks. Beyond the descriptive synthesis, the four-tier LMIC feasibility classification system introduced in this review is intended as a reusable instrument: future researchers, funders, and health-system planners can apply it independently to characterize the deployment readiness of new AI tools in resource-constrained settings, regardless of disease domain. Priority future directions include prospective multi-centre LMIC validation, non-invasive endometriosis diagnostics, algorithmic fairness evaluation, and implementation science — all oriented toward ensuring that AI progress in reproductive health reaches the women who bear the greatest unmet burden. Consequently, the findings of this review reflect trends predominantly from East Asian and high-income settings and should not be interpreted as globally generalizable. Translational recommendations for LMICs remain theoretical until validated in local, resource-constrained populations. These conclusions should be interpreted within the descriptive scope of scoping review methodology and not as causal or comparative inferences.

## Supporting information

S1 File PRISMA-ScR Checklist. Completed PRISMA-ScR checklist covering all 20 essential items and 2 optional items.
(DOCX)

S1 Table Risk-of-bias scoring rubric. Explicit domain-specific criteria used to classify studies as low, moderate, or high risk of bias (adapted from PROBAST-AI, QUADAS-AI, and TRIPOD-AI).
(DOCX)

S2 Table LMIC applicability categorical criteria. Full definitions of the four LMIC-feasibility categories (High, Moderate, Low, Very Low) used to classify studies.
(DOCX)
