# Supplementary material for: Artificial intelligence for women’s reproductive health: A scoping review of global diagnostic trends, methodological gaps, and a translational research agenda for low-resource settings
Source: PLoS One. 2026 Sep 25;21(9):e0358557. doi: 10.1371/journal.pone.0358557 (PMC13614794; doi:10.1371/journal.pone.0358557)
Supplement: Supplementary file 2 [file pone.0358557.s002.docx]

**S2 Table. Risk-of-Bias Scoring Rubric.**

Explicit domain-specific criteria used to classify studies as low, moderate, or high risk of bias (adapted from PROBAST-AI, QUADAS-AI, and TRIPOD-AI).

**Purpose and Application**

This rubric specifies the explicit criteria used to classify each of the 116 included studies as being at Low, Moderate, or High risk of bias across five methodological domains. The rubric was adapted from PROBAST-AI, QUADAS-AI, and TRIPOD-AI and applied consistently by two reviewers (MMHM and TI), with discrepancies resolved by a third reviewer (TS).

**Overall Risk Classification Logic**

- Low Risk: Study meets Low Risk criteria in all five domains.
- Moderate Risk: Study meets High Risk criteria in 1 or 2 domains.
- High Risk: Study meets High Risk criteria in 3 or more domains.

Studies falling between Low and High thresholds in a given domain were classified as Moderate for that domain but counted as Low Risk in the overall classification logic above.

| **Domain** | **Criterion for LOW Risk** | **Criterion for HIGH Risk** |
| --- | --- | --- |
| 1. Participant Selection | Multi-centre recruitment AND sample size justification provided AND >500 participants | Single-centre AND convenience sampling AND no sample size justification |
| 2. Predictor Measurement | Prospective data collection OR blinded retrospective extraction with documented protocol | Retrospective extraction without blinding AND inconsistent feature engineering |
| 3. Outcome Assessment | Reference standard blinded to AI prediction AND surgical/pathological confirmation available | Reference standard not blinded AND no surgical confirmation for negative cases |
| 4. Model Development | Hyperparameter tuning documented AND class imbalance addressed AND no feature selection leakage | No documentation of tuning AND class imbalance unaddressed AND feature selection before CV splitting |
| 5. Model Validation | External validation on independent dataset AND temporal validation performed | No external validation AND no temporal validation AND validation set <20% of total |

**Distribution Across Included Studies (n = 116)**

The distribution of studies across risk levels for each domain is reported in Table 10 of the main manuscript. Overall, 83.6% of studies were classified as being at high risk of bias.

**Inter-rater Agreement**

Cohen’s κ for risk-of-bias classification on a 20% random subsample (n = 23 studies) was κ = 0.81 (substantial agreement). The primary sources of disagreement were the Predictor Measurement and Model Development domains.
