# Supplementary material for: Artificial intelligence for women’s reproductive health: A scoping review of global diagnostic trends, methodological gaps, and a translational research agenda for low-resource settings
Source: PLoS One. 2026 Sep 25;21(9):e0358557. doi: 10.1371/journal.pone.0358557 (PMC13614794; doi:10.1371/journal.pone.0358557)
Supplement: Supplementary file 3 [file pone.0358557.s003.docx]

**S3 Table. LMIC Applicability Categorical Criteria.**

Full definitions of the four LMIC-feasibility categories (High, Moderate, Low, Very Low) used to classify studies.

## **Rationale**

Each included study was evaluated for deployment feasibility in LMIC settings using a four-tier qualitative classification scheme defined prospectively. A categorical scheme was preferred over a continuous numeric score to avoid conveying unwarranted precision, to improve reproducibility across reviewers, and to better reflect the threshold-based nature of real-world infrastructure constraints.

## **Category Definitions**

| **Category** | **Infrastructure Required** | **Personnel** | **Cost/Test** | **Deployment Level** |
| --- | --- | --- | --- | --- |
| High Feasibility | Basic clinical variables only (age, BMI, BP, symptom questionnaires) | No specialist required; nurse or community health worker | <$10 | Primary care / community setting |
| Moderate Feasibility | Routine laboratory tests or basic ultrasound | Nurse or midwife-level personnel | $10–25 | Secondary care / antenatal clinic |
| Low Feasibility | Specialist imaging (standard ultrasound with trained radiologist) or advanced laboratory panels | Specialist personnel (radiologist, gynaecologist) | $25–50 | District hospital |
| Very Low Feasibility | MRI, advanced imaging, or multi-omics platforms | Highly specialized personnel (subspecialists, laboratory scientists) | >$50 | Tertiary centre only |

## **Assignment Rule**

Each study was assigned to the least favourable category among its required inputs. For example, a study combining basic clinical variables with MRI would be classified as “Very Low Feasibility” because MRI is the limiting factor. Where a study evaluated multiple model configurations, classification was based on the best-performing configuration that the authors recommended for deployment.

## **Example Mapping**

| **Study** | **Clinical Focus & Inputs** | **Setting** | **Category** |
| --- | --- | --- | --- |
| [22] | Osteopenia detection; BIA device, age, BMI | Primary care | Moderate Feasibility |
| [25] | Hypertension screening; BP cuff, waist circumference tape | Community health | High Feasibility |
| [27] | Endometriosis screening; clinical history and symptoms only | Primary care | High Feasibility |
| [66] | Endometriosis detection; electrochemical biosensor | Primary care | High Feasibility |
| [45] | Endometriosis detection; urine infrared spectrometry | Secondary care | Moderate Feasibility |
| [75] | GDM prediction; first-trimester routine labs | Antenatal clinic | Moderate Feasibility |
| [32] | Uterine fibroid diagnosis; ultrasound + radiologist interpretation | District hospital | Low Feasibility |
| [119] | PCOS diagnosis; ultrasound + radiologist | Tertiary centre | Low Feasibility |
| [36] | Uterine fibroid embolisation prediction; MRI features | Tertiary centre | Very Low Feasibility |
| [123] | PCOS gene association; transcriptomic multi-omics | Tertiary / research | Very Low Feasibility |

## **Relationship to Resource Tiers**

The LMIC Feasibility categories align with the Resource Tiers used in Tables 6 and 7 of the main manuscript as follows:

• Tier 1 (Low resource) ≈ High Feasibility

• Tier 2 (Medium resource) ≈ Moderate Feasibility

• Tier 2–3 (Medium–High resource with specialist imaging) ≈ Low Feasibility

• Tier 3 (High resource) ≈ Very Low Feasibility
