# Supplementary material for: Artificial intelligence for women’s reproductive health: A scoping review of global diagnostic trends, methodological gaps, and a translational research agenda for low-resource settings
Source: PLoS One. 2026 Sep 25;21(9):e0358557. doi: 10.1371/journal.pone.0358557 (PMC13614794; doi:10.1371/journal.pone.0358557)
Supplement: Supplementary file 4 [file pone.0358557.s004.docx]

**Response to Academic Editor and Reviewers**

**Manuscript Title:** Artificial intelligence for women's reproductive health: A scoping review of global diagnostic trends, methodological gaps, and a translational research agenda for low-resource settings

**Journal: PLOS ONE**

**Manuscript Number:** PONE-D-26-22995

**Dear Dr. Kwang-Sig Lee and Reviewers,**

We sincerely thank the Academic Editor and the two reviewers for their thoughtful and constructive feedback on our manuscript. We have carefully addressed all comments and have revised the manuscript accordingly. Below we provide a point-by-point response to each concern raised.

# RESPONSE TO ACADEMIC EDITOR'S JOURNAL REQUIREMENTS

**Comment 1:** "Please ensure that your manuscript meets PLOS ONE's style requirements, including those for file naming."

**Response:** We thank the Editor for this guidance and confirm that the manuscript now fully complies with all PLOS ONE style and file naming requirements. We have ensured that all main figures and supporting information files are saved and uploaded individually with the correct naming conventions (e.g., Fig1.tif, S1_File.pdf). Within the manuscript, we corrected all figure citations to remove periods (e.g., "Fig 1"), standardized supporting information citations to the required shorthand (e.g., "S1 File"), updated section headings to match PLOS standards (e.g., "Materials and methods", "Conclusions"), and corrected the equal contribution symbols and corresponding author email format on the title page.

**Comment 2:** "Please ensure that you have uploaded your PRISMA flowchart as Figure 1."

**Response:** We confirm that the PRISMA flow diagram is designated as Figure 1 in the manuscript text. Additionally, the corresponding image file has been uploaded separately to the submission portal and named exactly as "Fig1.tif" in strict accordance with PLOS ONE's file naming guidelines.

**Comment 3:** "We note that Figure 5 in your submission contains [map/satellite] images which may be copyrighted. All PLOS content is published under the Creative Commons Attribution License (CC BY 4.0)... We require you to either (1) present written permission from the copyright holder to publish these figures specifically under the CC BY 4.0 license, or (2) remove the figures from your submission."

**Response:** We thank the Editor for bringing this copyright issue to our attention. We have removed the copyrighted map/satellite images from our submission and replaced them with a newly created, original figure titled "Geographic Distribution and Key Methodological Gaps in AI-Assisted Medical Imaging Research." As the sole creators of this new figure, we hold full copyright and explicitly consent to publish it under the CC BY 4.0 license. All relevant figure citations, captions, and numbering in the manuscript have been updated to reflect this change.

**Comment 4:** "Please remove your figures from within your manuscript file, leaving only the individual TIFF/EPS image files, uploaded separately."

**Response:** We have complied with this requirement. Since our manuscript is prepared using LaTeX, we have retained only the \includegraphics references in the .tex source file (as permitted by PLOS ONE's policy for LaTeX submissions) and have uploaded all seven figures as separate high-resolution TIFF files (≥300 dpi) through the submission system. All figure captions remain in the manuscript file.

**Comment 5:** "Please include captions for your Supporting Information files at the end of your manuscript, and update any in-text citations to match accordingly."

**Response:** We have added a dedicated 'Supporting information' section at the end of our manuscript (after the References) containing complete captions for all supporting files. The section includes: S1 File (PRISMA-ScR Checklist), S2 Table (Risk-of-Bias Scoring Rubric), and S3 Table (LMIC Applicability Categorical Criteria). All in-text citations have been updated to use PLOS ONE's standard format (e.g., 'S1 File', 'S2 Table', 'S3 Table') instead of 'Supplementary File S1' or 'Supplementary Table S2'

**Comment 6:** "If the reviewer comments include a recommendation to cite specific previously published works, please review and evaluate these publications to determine whether they are relevant and should be cited."

**Response:** We have carefully reviewed and incorporated all citation recommendations from the reviewers. Specifically:

- SHAP (Reviewer #2, Comment 2): Added the official web address and primary citation (Lundberg & Lee, 2017)
- OpenHIE and WHO SMART Guidelines (Reviewer #2, Comment 9): Added complete references with supporting citations
- Supporting citations for Lines 456-459 (Reviewer #2, Comment 10): Added references for AI cost-effectiveness in LMICs, mobile-based AI deployment, and implementation science frameworks

All recommended citations have been evaluated as relevant and have been included in the revised manuscript and reference list.

# Reviewer 1

**Comment 1:** "The authors should make clear whether the review follows PRISMA-ScR and should provide a completed checklist if applicable."

**Response:** We thank the reviewer for this clarification. We confirm that this scoping review strictly follows the PRISMA-ScR (Preferred Reporting Items for Systematic Reviews and Meta-Analyses Extension for Scoping Reviews) guidelines. We have explicitly stated our adherence to these guidelines in Section 2 (Materials and methods). Furthermore, as requested, we have provided the fully completed PRISMA-ScR checklist as S1 File in the "Supporting information" section at the end of the manuscript.

**Comment 2:** "The search strategy should be fully reproducible, including databases searched, search dates, complete search strings, language restrictions, handling of grey literature and preprints, and the full inclusion/exclusion criteria."

**Response:** We thank the reviewer for this important methodological clarification. We have revised Section 2.2 (Search Strategy) to explicitly state the exact search dates (December 2025 to February 2026), language restrictions (English-only), and our handling of grey literature and preprints (which were explicitly excluded). The complete, reproducible search strings for all five databases are detailed in Table 1 and provided in full in the newly added Supplementary File S4. Supplementary file S4 excel file contains 18 columns which was not possible to include in main manuscript, so we have added this as extra supplementary file . The full inclusion and exclusion criteria remain comprehensively detailed in Section 2.3 (Eligibility Criteria).

**Comment 3:** "The screening and extraction workflow also requires clearer reporting. The authors should specify how many reviewers screened records and extracted data, how disagreements were resolved, and what variables were extracted from each included study."

**Response:** We appreciate this feedback and have significantly strengthened the reporting of our screening and extraction workflows in Sections 2.4 and 2.5. We have clarified that the screening workflow involved two reviewers (MMHM and TI) for title/abstract screening and two reviewers (AFS and FS) for full-text screening, with discrepancies resolved through consensus with a third reviewer (TS). Furthermore, we have explicitly listed all extracted variables across six specific domains in Section 2.5 and detailed our rigorous, multi-tier independent verification process: initial extraction by one reviewer (TI), second-stage verification by three reviewers (OF, AFS, and FS), and final third-stage verification by MMHM. We also clarified that a 20% random sample was cross verified, yielding a high inter-rater agreement (Cohen’s κ = 0.92), with any remaining discrepancies resolved by the senior authors (RUI and TS).

**Comment 4:** "Any descriptive counts, proportions, diagnostic categories, AI model categories, or geographic summaries should be tied to a transparent extraction dataset with clear denominators."

**Response:** We agree with the reviewer that clear denominators are essential for transparency. We have added a clarifying statement at the beginning of Section 2.7 (Data Synthesis and Reporting) and Section 3 (Results) explicitly defining the denominators. All proportions, percentages, and descriptive counts are now clearly tied to the total denominator of N = 116 included studies, unless a specific subgroup denominator is explicitly stated in the respective tables or text. Furthermore, the full transparent extraction dataset, which allows readers to verify all counts and proportions, is now available in Supplementary File S4.

**Comment 5:** "The conclusions should be narrowed where necessary. Claims about 'global' trends and translational relevance for low-resource settings must be directly supported by the geographic distribution, study settings, validation quality, and clinical context of the included studies. If the evidence base is concentrated in a limited number of countries or settings, this should be stated clearly and reflected in the conclusions."

**Response:** We thank the reviewer for this crucial observation. We have carefully revised the Abstract, Introduction, and Conclusion to narrow our claims and ensure they are strictly supported by the mapped evidence. The term "global diagnostic trends" has been changed to "international diagnostic trends across 27 countries." We have explicitly added limitations to the Conclusion regarding the heavy concentration of evidence in East Asia (39.6%) and high-income countries, and the severe underrepresentation of Sub-Saharan Africa (1.7%) and specific South Asian nations (e.g., Nepal, Sri Lanka). Translational claims for LMICs are now strictly qualified by the methodological limitations (e.g., 75.5% lack of external validation) and geographic gaps identified in our review.

**Comment 6:** "The data underlying the review should be made fully available. For a scoping review, this should include the full list of included studies, extracted study-level variables, coding/category definitions, search strategies, screening results, and the data used to generate all tables, figures, counts, and proportions."

**Response:** We have fully expanded our Data Availability Statement and updated our Mendeley Data repository to ensure complete transparency. The repository now contains: (1) the full list of 116 included studies with complete extracted study-level variables (Supplementary File S4); (2) the complete, reproducible search strategies for all five databases and the screening log detailing reasons for exclusion at each stage (Supplementary File S5); (3) coding and category definitions for the six taxonomy categories and LMIC feasibility tiers (Supplementary Tables S2 and S3); and (4) the raw data used to generate all tables, figures, counts, and proportions. Comment 7: "Overall, the manuscript has potential, but publication should depend on stronger methodological reporting, complete data availability, and conclusions that are more tightly linked to the mapped evidence."

**Comment 7:** "Overall, the manuscript has potential, but publication should depend on stronger methodological reporting, complete data availability, and conclusions that are more tightly linked to the mapped evidence."

**Response:** We thank the reviewer for their constructive evaluation and for recognizing the potential of our manuscript. We have comprehensively addressed all methodological reporting gaps, ensured complete data availability via our updated Mendeley Data repository, and carefully aligned our conclusions with the actual geographic and methodological distribution of the mapped evidence. We believe these revisions have significantly strengthened the manuscript's rigor, transparency, and overall quality.

# Reviewer 2

## Comment 1: "Line 96: Please provide the web address for Rayyan."

**Response:** We have added the official Rayyan web address and the primary citation to the reference list. The in-text citation now appears as references [20, 21] at Line 97. The web address is available in the reference entry and also through the citation link. Thank you for this helpful suggestion.

## Comment 2: "Line 237: Please provide the official web address or an appropriate reference for SHAP."

**Response:** We have added the primary citation for SHAP as reference [137] in the revised manuscript.

## Comment 3: "Figure 4: Please include a separate legend explaining the relative values represented by the bubble sizes. In addition, clearly describe how the LMIC feasibility threshold was calculated and how it was used to position the dashed line."

**Response:** We thank the reviewer for this important clarification request. We have revised Figure 4 to include:

1. A separate legend explains that bubble sizes represent the number of studies (with larger bubbles indicating more studies) for each combination of diagnostic category and AI methodology, while bubble colors represent the proportion of studies from LMIC settings.
2. The LMIC feasibility threshold (shown as a dashed line at 15%) was calculated as the 75th percentile of LMIC representation across all diagnostic categories. This threshold was chosen to distinguish categories with relatively higher LMIC representation from those with lower representation. We have validated this through sensitivity analysis using alternative cutoffs (10%, 20%, 25%), which produced consistent results. The dashed line visually separates diagnostic-AI combinations that meet the threshold from those that do not.

## Comment 4: "Table 9: Please define the abbreviation 'SAARC Avg.'"

**Response:** We have defined "SAARC Avg." as "South Asian Association for Regional Cooperation Average" in table footnote.

## Comment 5: "Figure 5A: What does the orange color represent? Please clarify this in the figure legend."

## Comment 6: "Figure 5B: Please explain the meaning of the colors and include an appropriate legend."

**Response for Comments 5 & 6:** We thank the reviewer for this query. Please note that the original Figure 5 (which contained map/satellite images) was removed and replaced in accordance with a specific request from the Editorial Office regarding copyright restrictions under the CC BY 4.0 license. It has been replaced with a new, original figure (now designated as Figure [insert new figure number, e.g., 5]) that illustrates the geographic distribution and methodological gaps without using any copyrighted imagery. Consequently, the specific sub-panels 5A and 5B from the original submission are no longer present in the revised manuscript, and the new figure includes a comprehensive, self-explanatory legend.

## Comment 7: "Table 12 and Figure 7: These appear to present the same data. This duplication is unnecessary; therefore, I suggest removing Table 12."

**Response:** We have Removed Table 12 as suggested, table 13 has now in place of table 12.

## Comment 8: "Line 334: The abbreviation LMIC has already been defined earlier in the manuscript. Please avoid defining it again."

**Response:**  We have removed the duplicate definition at Line 334. LMIC is now consistently defined only at its first occurrence in tables 2’s footnote . We have reviewed the entire manuscript to eliminate any other redundant definitions.

## Comment 9: "Line 435: Please provide supporting citations and complete references for OpenHIE and WHO SMART Guidelines."

**Response:** We have added complete references for OpenHIE and WHO SMART Guidelines in the revised manuscript. The citations [138] and [139] now reference the OpenHIE architecture framework, and citation [140] references the WHO SMART Guidelines methodology. These references are included in the reference list with full bibliographic details as requested.

## Comment 10: "Lines 456–459: Please include at least one supporting citation for these statements."

**Response:** We have added supporting citations to the statements regarding imaging model performance decay and domain adaptation methods in the revised manuscript (Lines 456-469). The added references [141-144] now support the claims about performance decay in imaging-based models across different scanners and protocols, as well as domain adaptation techniques including histogram matching, CycleGAN-based style transfer, and test-time adaptation approaches demonstrated in comparable imaging tasks such as mammography and chest radiography. These citations provide the necessary evidence for the statements identified by the reviewer.
