# Supplementary material for: Artificial intelligence for women’s reproductive health: A scoping review of global diagnostic trends, methodological gaps, and a translational research agenda for low-resource settings
Source: PLoS One. 2026 Sep 25;21(9):e0358557. doi: 10.1371/journal.pone.0358557 (PMC13614794; doi:10.1371/journal.pone.0358557)
Supplement: Peer review history

# Peer review: Decision Letter 0

**DOI:** 10.1371/journal.pone.0358557.r001

**Authors:** Kwang-Sig Lee

21 Jul 2026

Dear Dr. Munna,

Thank you for submitting your manuscript to PLOS ONE. After careful consideration, we feel that it has merit but does not fully meet PLOS ONE’s publication criteria as it currently stands. Therefore, we invite you to submit a revised version of the manuscript that addresses the points raised during the review process.

Please submit your revised manuscript by Sep 19 2026 11:59PM. If you will need more time than this to complete your revisions, please reply to this message or contact the journal office at plosone@plos.org. When you're ready to submit your revision, log on to https://www.editorialmanager.com/pone/ and select the 'Submissions Needing Revision' folder to locate your manuscript file.

- A letter that responds to each point raised by the academic editor and reviewer(s). You should upload this letter as a separate file labeled 'Response to Reviewers'.
- A marked-up copy of your manuscript that highlights changes made to the original version. You should upload this as a separate file labeled 'Revised Manuscript with Track Changes'.
- An unmarked version of your revised paper without tracked changes. You should upload this as a separate file labeled 'Manuscript'.

If you would like to make changes to your financial disclosure, please include your updated statement in your cover letter. Guidelines for resubmitting your figure files are available below the reviewer comments at the end of this letter.

If applicable, we recommend that you deposit your laboratory protocols in protocols.io to enhance the reproducibility of your results. Protocols.io assigns your protocol its own identifier (DOI) so that it can be cited independently in the future. For instructions see: https://journals.plos.org/plosone/s/submission-guidelines#loc-laboratory-protocols. Additionally, PLOS ONE offers an option for publishing peer-reviewed Lab Protocol articles, which describe protocols hosted on protocols.io. Read more information on sharing protocols at https://plos.org/protocols?utm_medium=editorial-email&utm_source=authorletters&utm_campaign=protocols.

As the corresponding author, your ORCID iD is verified in the submission system and will appear in the published article. PLOS supports the use of ORCID, and we encourage all coauthors to register for an ORCID iD and use it as well. Please encourage your coauthors to verify their ORCID iD within the submission system before final acceptance, as unverified ORCID iDs will not appear in the published article. *Only*  the individual author can complete the verification step; PLOS staff *cannot*  verify ORCID iDs on behalf of authors.

We look forward to receiving your revised manuscript.

Kind regards,

Kwang-Sig Lee

Academic Editor

PLOS One

Journal Requirements:

When submitting your revision, we need you to address these additional requirements.

1. Please ensure that your manuscript meets PLOS ONE's style requirements, including those for file naming. The PLOS ONE style templates can be found at

https://journals.plos.org/plosone/s/file?id=wjVg/PLOSOne_formatting_sample_main_body.pdf and

https://journals.plos.org/plosone/s/file?id=ba62/PLOSOne_formatting_sample_title_authors_affiliations.pdf.

2. Please ensure that you have uploaded your PRISMA flowchart as Figure 1, or else explain why this is not possible. Blank flowcharts can be found here: http://www.prisma-statement.org/

3. We note that Figure 5 in your submission contain [map/satellite] images which may be copyrighted. All PLOS content is published under the Creative Commons Attribution License (CC BY 4.0), which means that the manuscript, images, and Supporting Information files will be freely available online, and any third party is permitted to access, download, copy, distribute, and use these materials in any way, even commercially, with proper attribution. For these reasons, we cannot publish previously copyrighted maps or satellite images created using proprietary data, such as Google software (Google Maps, Street View, and Earth). For more information, see our copyright guidelines: http://journals.plos.org/plosone/s/licenses-and-copyright.

We require you to either (1) present written permission from the copyright holder to publish these figures specifically under the CC BY 4.0 license, or (2) remove the figures from your submission:

a. You may seek permission from the original copyright holder of Figure 5 to publish the content specifically under the CC BY 4.0 license.

We recommend that you contact the original copyright holder with the Content Permission Form (http://journals.plos.org/plosone/s/file?id=7c09/content-permission-form.pdf) and the following text:

“I request permission for the open-access journal PLOS ONE to publish XXX under the Creative Commons Attribution License (CCAL) CC BY 4.0 (http://creativecommons.org/licenses/by/4.0/). Please be aware that this license allows unrestricted use and distribution, even commercially, by third parties. Please reply and provide explicit written permission to publish XXX under a CC BY license and complete the attached form.”

Please upload the completed Content Permission Form or other proof of granted permissions as an "Other" file with your submission.

In the figure caption of the copyrighted figure, please include the following text: “Reprinted from [ref] under a CC BY license, with permission from [name of publisher], original copyright [original copyright year].”

b. If you are unable to obtain permission from the original copyright holder to publish these figures under the CC BY 4.0 license or if the copyright holder’s requirements are incompatible with the CC BY 4.0 license, please either i) remove the figure or ii) supply a replacement figure that complies with the CC BY 4.0 license. Please check copyright information on all replacement figures and update the figure caption with source information. If applicable, please specify in the figure caption text when a figure is similar but not identical to the original image and is therefore for illustrative purposes only.

The following resources for replacing copyrighted map figures may be helpful:

USGS National Map Viewer (public domain): http://viewer.nationalmap.gov/viewer/

The Gateway to Astronaut Photography of Earth (public domain): http://eol.jsc.nasa.gov/sseop/clickmap/

Maps at the CIA (public domain): https://www.cia.gov/library/publications/the-world-factbook/index.html and https://www.cia.gov/library/publications/cia-maps-publications/index.html

NASA Earth Observatory (public domain): http://earthobservatory.nasa.gov/

Landsat: http://landsat.visibleearth.nasa.gov/

USGS EROS (Earth Resources Observatory and Science (EROS) Center) (public domain): http://eros.usgs.gov/#

Natural Earth (public domain): http://www.naturalearthdata.com/

4. Please remove your figures from within your manuscript file, leaving only the individual TIFF/EPS image files, uploaded separately. These will be automatically included in the reviewers’ PDF."

5. Please include captions for your Supporting Information files at the end of your manuscript, and update any in-text citations to match accordingly. Please see our Supporting Information guidelines for more information: http://journals.plos.org/plosone/s/supporting-information.

6. If the reviewer comments include a recommendation to cite specific previously published works, please review and evaluate these publications to determine whether they are relevant and should be cited. There is no requirement to cite these works unless the editor has indicated otherwise.

[Note: HTML markup is below. Please do not edit.]

Reviewers' comments:

Reviewer's Responses to Questions

**Comments to the Author**

1. Is the manuscript technically sound, and do the data support the conclusions?

Reviewer #1: Partly

Reviewer #2: Yes

**********

2. Has the statistical analysis been performed appropriately and rigorously?

Reviewer #1: N/A

Reviewer #2: Yes

**********

3. Have the authors made all data underlying the findings in their manuscript fully available?

The PLOS Data policy

Reviewer #1: No

Reviewer #2: Yes

**********

4. Is the manuscript presented in an intelligible fashion and written in standard English?

Reviewer #1: Yes

Reviewer #2: Yes

**********

Reviewer #1: The manuscript addresses a timely and important topic: the use of artificial intelligence in women’s reproductive health, with attention to diagnostic trends, methodological gaps, and relevance for low-resource settings. The topic is suitable for PLOS ONE and the manuscript is generally intelligible and written in standard English.

However, I recommend major revision before publication. Because this is a scoping review, the main issue is not experimental replication or inferential statistics, but the transparency and reproducibility of the review methods. The authors should make clear whether the review follows PRISMA-ScR and should provide a completed checklist if applicable. The search strategy should be fully reproducible, including databases searched, search dates, complete search strings, language restrictions, handling of grey literature and preprints, and the full inclusion/exclusion criteria.

The screening and extraction workflow also requires clearer reporting. The authors should specify how many reviewers screened records and extracted data, how disagreements were resolved, and what variables were extracted from each included study. Any descriptive counts, proportions, diagnostic categories, AI model categories, or geographic summaries should be tied to a transparent extraction dataset with clear denominators.

The conclusions should be narrowed where necessary. Claims about “global” trends and translational relevance for low-resource settings must be directly supported by the geographic distribution, study settings, validation quality, and clinical context of the included studies. If the evidence base is concentrated in a limited number of countries or settings, this should be stated clearly and reflected in the conclusions.

Finally, the data underlying the review should be made fully available. For a scoping review, this should include the full list of included studies, extracted study-level variables, coding/category definitions, search strategies, screening results, and the data used to generate all tables, figures, counts, and proportions. If these are not already included as supporting information or in a public repository, they should be provided at revision.

Overall, the manuscript has potential, but publication should depend on stronger methodological reporting, complete data availability, and conclusions that are more tightly linked to the mapped evidence.

Reviewer #2: In the manuscript PONE-D-26-22995 “Artificial Intelligence for Women’s Reproductive Health: A Scoping Review of Global Diagnostic Trends, Methodological Gaps, and a Translational Research Agenda for Low-Resource Settings,” the authors conducted a PRISMA-ScR-guided scoping review of artificial intelligence (AI) and machine learning (ML) applications in women’s reproductive and hormonal health.

The objective and scope of the review are clearly defined and consistent throughout the manuscript. Overall, the manuscript is well written, the methods are described in sufficient detail, and the data appear to have been appropriately managed. The results are generally well presented, although some figures and tables require further clarification. The discussion is consistent with the reported findings, and the data generated in the study are publicly accessible.

The taxonomy developed for this review represents a valuable contribution. In addition, the differences in the types and extent of AI/ML applications across country-income categories are clearly described.

Minor comments

Line 96: Please provide the web address for Rayyan.

Line 237: Please provide the official web address or an appropriate reference for SHAP.

Figure 4: Please include a separate legend explaining the relative values represented by the bubble sizes. In addition, clearly describe how the LMIC feasibility threshold was calculated and how it was used to position the dashed line.

Table 9: Please define the abbreviation “SAARC Avg.”

Figure 5A: What does the orange color represent? Please clarify this in the figure legend.

Figure 5B: Please explain the meaning of the colors and include an appropriate legend.

Table 12 and Figure 7: These appear to present the same data. This duplication is unnecessary; therefore, I suggest removing Table 12.

Line 334: The abbreviation LMIC has already been defined earlier in the manuscript. Please avoid defining it again.

Line 435: Please provide supporting citations and complete references for OpenHIE and WHO SMART Guidelines.

Lines 456–459: Please include at least one supporting citation for these statements.

**********

what does this mean?). If published, this will include your full peer review and any attached files.

If you choose “no”, your identity will remain anonymous but your review may still be made public.

**Do you want your identity to be public for this peer review?**  For information about this choice, including consent withdrawal, please see our Privacy Policy

Reviewer #1: **Yes:**  Ulysses Angulo

Reviewer #2: No

**********

[NOTE: If reviewer comments were submitted as an attachment file, they will be attached to this email and accessible via the submission site. Please log into your account, locate the manuscript record, and check for the action link "View Attachments". If this link does not appear, there are no attachment files.]

To ensure your figures meet our technical requirements, please review our figure guidelines: https://journals.plos.org/plosone/s/figures

You may also use PLOS’s free figure tool, NAAS, to help you prepare publication quality figures: https://journals.plos.org/plosone/s/figures#loc-tools-for-figure-preparation.

NAAS will assess whether your figures meet our technical requirements by comparing each figure against our figure specifications.

# Author comment: Author response to Decision Letter 1

**DOI:** 10.1371/journal.pone.0358557.r002

31 Jul 2026

RESPONSE TO ACADEMIC EDITOR'S JOURNAL REQUIREMENTS

Comment 1: "Please ensure that your manuscript meets PLOS ONE's style requirements, including those for file naming."

Response: We thank the Editor for this guidance and confirm that the manuscript now fully complies with all PLOS ONE style and file naming requirements. We have ensured that all main figures and supporting information files are saved and uploaded individually with the correct naming conventions (e.g., Fig1.tif, S1_File.pdf). Within the manuscript, we corrected all figure citations to remove periods (e.g., "Fig 1"), standardized supporting information citations to the required shorthand (e.g., "S1 File"), updated section headings to match PLOS standards (e.g., "Materials and methods", "Conclusions"), and corrected the equal contribution symbols and corresponding author email format on the title page.

Comment 2: "Please ensure that you have uploaded your PRISMA flowchart as Figure 1."

Response: We confirm that the PRISMA flow diagram is designated as Figure 1 in the manuscript text. Additionally, the corresponding image file has been uploaded separately to the submission portal and named exactly as "Fig1.tif" in strict accordance with PLOS ONE's file naming guidelines.

Comment 3: "We note that Figure 5 in your submission contains [map/satellite] images which may be copyrighted. All PLOS content is published under the Creative Commons Attribution License (CC BY 4.0)... We require you to either (1) present written permission from the copyright holder to publish these figures specifically under the CC BY 4.0 license, or (2) remove the figures from your submission."

Response: We thank the Editor for bringing this copyright issue to our attention. We have removed the copyrighted map/satellite images from our submission and replaced them with a newly created, original figure titled "Geographic Distribution and Key Methodological Gaps in AI-Assisted Medical Imaging Research." As the sole creators of this new figure, we hold full copyright and explicitly consent to publish it under the CC BY 4.0 license. All relevant figure citations, captions, and numbering in the manuscript have been updated to reflect this change.

Comment 4: "Please remove your figures from within your manuscript file, leaving only the individual TIFF/EPS image files, uploaded separately."

Response: We have complied with this requirement. Since our manuscript is prepared using LaTeX, we have retained only the \includegraphics references in the .tex source file (as permitted by PLOS ONE's policy for LaTeX submissions) and have uploaded all seven figures as separate high-resolution TIFF files (≥300 dpi) through the submission system. All figure captions remain in the manuscript file.

Comment 5: "Please include captions for your Supporting Information files at the end of your manuscript, and update any in-text citations to match accordingly."

Response: We have added a dedicated 'Supporting information' section at the end of our manuscript (after the References) containing complete captions for all supporting files. The section includes: S1 File (PRISMA-ScR Checklist), S2 Table (Risk-of-Bias Scoring Rubric), and S3 Table (LMIC Applicability Categorical Criteria). All in-text citations have been updated to use PLOS ONE's standard format (e.g., 'S1 File', 'S2 Table', 'S3 Table') instead of 'Supplementary File S1' or 'Supplementary Table S2'

Comment 6: "If the reviewer comments include a recommendation to cite specific previously published works, please review and evaluate these publications to determine whether they are relevant and should be cited."

Response: We have carefully reviewed and incorporated all citation recommendations from the reviewers. Specifically:

• SHAP (Reviewer #2, Comment 2): Added the official web address and primary citation (Lundberg & Lee, 2017)

• OpenHIE and WHO SMART Guidelines (Reviewer #2, Comment 9): Added complete references with supporting citations

• Supporting citations for Lines 456-459 (Reviewer #2, Comment 10): Added references for AI cost-effectiveness in LMICs, mobile-based AI deployment, and implementation science frameworks

All recommended citations have been evaluated as relevant and have been included in the revised manuscript and reference list.

Reviewer 1

Comment 1: "The authors should make clear whether the review follows PRISMA-ScR and should provide a completed checklist if applicable."

Response: We thank the reviewer for this clarification. We confirm that this scoping review strictly follows the PRISMA-ScR (Preferred Reporting Items for Systematic Reviews and Meta-Analyses Extension for Scoping Reviews) guidelines. We have explicitly stated our adherence to these guidelines in Section 2 (Materials and methods). Furthermore, as requested, we have provided the fully completed PRISMA-ScR checklist as S1 File in the "Supporting information" section at the end of the manuscript.

Comment 2: "The search strategy should be fully reproducible, including databases searched, search dates, complete search strings, language restrictions, handling of grey literature and preprints, and the full inclusion/exclusion criteria."

Response: We thank the reviewer for this important methodological clarification. We have revised Section 2.2 (Search Strategy) to explicitly state the exact search dates (December 2025 to February 2026), language restrictions (English-only), and our handling of grey literature and preprints (which were explicitly excluded). The complete, reproducible search strings for all five databases are detailed in Table 1 and provided in full in the newly added Supplementary File S4. Supplementary file S4 excel file contains 18 columns which was not possible to include in main manuscript, so we have added this as extra supplementary file . The full inclusion and exclusion criteria remain comprehensively detailed in Section 2.3 (Eligibility Criteria).

Comment 3: "The screening and extraction workflow also requires clearer reporting. The authors should specify how many reviewers screened records and extracted data, how disagreements were resolved, and what variables were extracted from each included study."

Response: We appreciate this feedback and have significantly strengthened the reporting of our screening and extraction workflows in Sections 2.4 and 2.5. We have clarified that the screening workflow involved two reviewers (MMHM and TI) for title/abstract screening and two reviewers (AFS and FS) for full-text screening, with discrepancies resolved through consensus with a third reviewer (TS). Furthermore, we have explicitly listed all extracted variables across six specific domains in Section 2.5 and detailed our rigorous, multi-tier independent verification process: initial extraction by one reviewer (TI), second-stage verification by three reviewers (OF, AFS, and FS), and final third-stage verification by MMHM. We also clarified that a 20% random sample was cross verified, yielding a high inter-rater agreement (Cohen’s κ = 0.92), with any remaining discrepancies resolved by the senior authors (RUI and TS).

Comment 4: "Any descriptive counts, proportions, diagnostic categories, AI model categories, or geographic summaries should be tied to a transparent extraction dataset with clear denominators."

Response: We agree with the reviewer that clear denominators are essential for transparency. We have added a clarifying statement at the beginning of Section 2.7 (Data Synthesis and Reporting) and Section 3 (Results) explicitly defining the denominators. All proportions, percentages, and descriptive counts are now clearly tied to the total denominator of N = 116 included studies, unless a specific subgroup denominator is explicitly stated in the respective tables or text. Furthermore, the full transparent extraction dataset, which allows readers to verify all counts and proportions, is now available in Supplementary File S4.

Comment 5: "The conclusions should be narrowed where necessary. Claims about 'global' trends and translational relevance for low-resource settings must be directly supported by the geographic distribution, study settings, validation quality, and clinical context of the included studies. If the evidence base is concentrated in a limited number of countries or settings, this should be stated clearly and reflected in the conclusions."

Response: We thank the reviewer for this crucial observation. We have carefully revised the Abstract, Introduction, and Conclusion to narrow our claims and ensure they are strictly supported by the mapped evidence. The term "global diagnostic trends" has been changed to "international diagnostic trends across 27 countries." We have explicitly added limitations to the Conclusion regarding the heavy concentration of evidence in East Asia (39.6%) and high-income countries, and the severe underrepresentation of Sub-Saharan Africa (1.7%) and specific South Asian nations (e.g., Nepal, Sri Lanka). Translational claims for LMICs are now strictly qualified by the methodological limitations (e.g., 75.5% lack of external validation) and geographic gaps identified in our review.

Comment 6: "The data underlying the review should be made fully available. For a scoping review, this should include the full list of included studies, extracted study-level variables, coding/category definitions, search strategies, screening results, and the data used to generate all tables, figures, counts, and proportions."

Response: We have fully expanded our Data Availability Statement and updated our Mendeley Data repository to ensure complete transparency. The repository now contains: (1) the full list of 116 included studies with complete extracted study-level variables (Supplementary File S4); (2) the complete, reproducible search strategies for all five databases and the screening log detailing reasons for exclusion at each stage (Supplementary File S5); (3) coding and category definitions for the six taxonomy categories and LMIC feasibility tiers (Supplementary Tables S2 and S3); and (4) the raw data used to generate all tables, figures, counts, and proportions. Comment 7: "Overall, the manuscript has potential, but publication should depend on stronger methodological reporting, complete data availability, and conclusions that are more tightly linked to the mapped evidence."

Comment 7: "Overall, the manuscript has potential, but publication should depend on stronger methodological reporting, complete data availability, and conclusions that are more tightly linked to the mapped evidence."

Response: We thank the reviewer for their constructive evaluation and for recognizing the potential of our manuscript. We have comprehensively addressed all methodological reporting gaps, ensured complete data availability via our updated Mendeley Data repository, and carefully aligned our conclusions with the actual geographic and methodological distribution of the mapped evidence. We believe these revisions have significantly strengthened the manuscript's rigor, transparency, and overall quality.

Reviewer 2

Comment 1: "Line 96: Please provide the web address for Rayyan."

Response: We have added the official Rayyan web address and the primary citation to the reference list. The in-text citation now appears as references [20, 21] at Line 97. The web address is available in the reference entry and also through the citation link. Thank you for this helpful suggestion.

Comment 2: "Line 237: Please provide the official web address or an appropriate reference for SHAP."

Response: We have added the primary citation for SHAP as reference [137] in the revised manuscript.

Comment 3: "Figure 4: Please include a separate legend explaining the relative values represented by the bubble sizes. In addition, clearly describe how the LMIC feasibility threshold was calculated and how it was used to position the dashed line."

Response: We thank the reviewer for this important clarification request. We have revised Figure 4 to include:

1. A separate legend explains that bubble sizes represent the number of studies (with larger bubbles indicating more studies) for each combination of diagnostic category and AI methodology, while bubble colors represent the proportion of studies from LMIC settings.

2. The LMIC feasibility threshold (shown as a dashed line at 15%) was calculated as the 75th percentile of LMIC representation across all diagnostic categories. This threshold was chosen to distinguish categories with relatively higher LMIC representation from those with lower representation. We have validated this through sensitivity analysis using alternative cutoffs (10%, 20%, 25%), which produced consistent results. The dashed line visually separates diagnostic-AI combinations that meet the threshold from those that do not.

Comment 4: "Table 9: Please define the abbreviation 'SAARC Avg.'"

Response: We have defined "SAARC Avg." as "South Asian Association for Regional Cooperation Average" in table footnote.

Comment 5: "Figure 5A: What does the orange color represent? Please clarify this in the figure legend."

Comment 6: "Figure 5B: Please explain the meaning of the colors and include an appropriate legend."

Response for Comments 5 & 6: We thank the reviewer for this query. Please note that the original Figure 5 (which contained map/satellite images) was removed and replaced in accordance with a specific request from the Editorial Office regarding copyright restrictions under the CC BY 4.0 license. It has been replaced with a new, original figure (now designated as Figure [insert new figure number, e.g., 5]) that illustrates the geographic distribution and methodological gaps without using any copyrighted imagery. Consequently, the specific sub-panels 5A and 5B from the original submission are no longer present in the revised manuscript, and the new figure includes a comprehensive, self-explanatory legend.

Comment 7: "Table 12 and Figure 7: These appear to present the same data. This duplication is unnecessary; therefore, I suggest removing Table 12."

Response: We have Removed Table 12 as suggested, table 13 has now in place of table 12.

Comment 8: "Line 334: The abbreviation LMIC has already been defined earlier in the manuscript. Please avoid defining it again."

Response: We have removed the duplicate definition at Line 334. LMIC is now consistently defined only at its first occurrence in tables 2’s footnote . We have reviewed the entire manuscript to eliminate any other redundant definitions.

Comment 9: "Line 435: Please provide supporting citations and complete references for OpenHIE and WHO SMART Guidelines."

Response: We have added complete references for OpenHIE and WHO SMART Guidelines in the revised manuscript. The citations [138] and [139] now reference the OpenHIE architecture framework, and citation [140] references the WHO SMART Guidelines methodology. These references are included in the reference list with full bibliographic details as requested.

Comment 10: "Lines 456–459: Please include at least one supporting citation for these statements."

Response: We have added supporting citations to the statements regarding imaging model performance decay and domain adaptation methods in the revised manuscript (Lines 456-469). The added references [141-144] now support the claims about performance decay in imaging-based models across different scanners and protocols, as well as domain adaptation techniques including histogram matching, CycleGAN-based style transfer, and test-time adaptation approaches demonstrated in comparable imaging tasks such as mammography and chest radiography. These citations provide the necessary evidence for the statements identified by the reviewer.

Attachment Submitted filename: PONE-D-26-22995_Response_to_Reviewers.docx

# Peer review: Decision Letter 1

**DOI:** 10.1371/journal.pone.0358557.r003

**Authors:** Kwang-Sig Lee

2 Sep 2026

Artificial intelligence for women’s reproductive health: A scoping review of global diagnostic trends, methodological gaps, and a translational research agenda for low-resource settings

PONE-D-26-22995R1

Dear Dr. Munna,

We’re pleased to inform you that your manuscript has been judged scientifically suitable for publication and will be formally accepted for publication once it meets all outstanding technical requirements.

Within one week, you’ll receive an e-mail detailing the required amendments. When these have been addressed, you’ll receive a formal acceptance letter and your manuscript will be scheduled for publication.

An invoice will be generated when your article is formally accepted. Please note, if your institution has a publishing partnership with PLOS and your article meets the relevant criteria, all or part of your publication costs will be covered. Please make sure your user information is up-to-date by logging into Editorial Manager at Editorial Manager® and clicking the ‘Update My Information' link at the top of the page. For questions related to billing, please contact billing support.

If your institution or institutions have a press office, please notify them about your upcoming paper to help maximize its impact. If they’ll be preparing press materials, please inform our press team as soon as possible -- no later than 48 hours after receiving the formal acceptance. Your manuscript will remain under strict press embargo until 2 pm Eastern Time on the date of publication. For more information, please contact onepress@plos.org.

Kind regards,

Kwang-Sig Lee

Academic Editor

PLOS One

Additional Editor Comments (optional):

Reviewers' comments:

Reviewer's Responses to Questions

**Comments to the Author**

Reviewer #2: All comments have been addressed

**********

2. Is the manuscript technically sound, and do the data support the conclusions?

Reviewer #2: Yes

**********

3. Has the statistical analysis been performed appropriately and rigorously?

Reviewer #2: Yes

**********

4. Have the authors made all data underlying the findings in their manuscript fully available?

The PLOS Data policy

Reviewer #2: Yes

**********

5. Is the manuscript presented in an intelligible fashion and written in standard English?

Reviewer #2: Yes

**********

Reviewer #2: (No Response)

**********

what does this mean?). If published, this will include your full peer review and any attached files.

If you choose “no”, your identity will remain anonymous but your review may still be made public.

**Do you want your identity to be public for this peer review?**  For information about this choice, including consent withdrawal, please see our Privacy Policy

Reviewer #2: No

**********

# Editor report: Acceptance letter

**DOI:** 10.1371/journal.pone.0358557.r004

**Authors:** Kwang-Sig Lee

PONE-D-26-22995R1

PLOS One

Dear Dr. Munna,

I'm pleased to inform you that your manuscript has been deemed suitable for publication in PLOS One. Congratulations! Your manuscript is now being handed over to our production team.

At this stage, our production department will prepare your paper for publication. This includes ensuring the following:

* All references, tables, and figures are properly cited

* All relevant supporting information is included in the manuscript submission,

* There are no issues that prevent the paper from being properly typeset

You will receive further instructions from the production team, including instructions on how to review your proof when it is ready. Please keep in mind that we are working through a large volume of accepted articles, so please give us a few days to review your paper and let you know the next and final steps.

Lastly, if your institution or institutions have a press office, please let them know about your upcoming paper now to help maximize its impact. If they'll be preparing press materials, please inform our press team within the next 48 hours. Your manuscript will remain under strict press embargo until 2 pm Eastern Time on the date of publication. For more information, please contact onepress@plos.org.

You will receive an invoice from PLOS for your publication fee after your manuscript has reached the completed accept phase. If you receive an email requesting payment before acceptance or for any other service, this may be a phishing scheme. Learn how to identify phishing emails and protect your accounts at https://explore.plos.org/phishing.

If we can help with anything else, please email us at customercare@plos.org.

Thank you for submitting your work to PLOS One and supporting open access.

Kind regards,

PLOS One Editorial Office Staff

on behalf of

Professor Kwang-Sig Lee

Academic Editor

PLOS One
